# Transcriptional and Translational Landscape of Equine Torovirus

**DOI:** 10.1128/JVI.00589-18

**Published:** 2018-08-16

**Authors:** Hazel Stewart, Katherine Brown, Adam M. Dinan, Nerea Irigoyen, Eric J. Snijder, Andrew E. Firth

**Affiliations:** a Division of Virology, Department of Pathology, University of Cambridge, Cambridge, United Kingdom; b Molecular Virology Laboratory, Department of Medical Microbiology, Leiden University Medical Center, Leiden, The Netherlands; University of Texas Southwestern Medical Center

**Keywords:** nidovirus, coronavirus, ribosomes, transcription, veterinary pathogens

## Abstract

The genus Torovirus (subfamily Torovirinae, family Coronaviridae, order Nidovirales) encompasses a range of species that infect domestic ungulates, including cattle, sheep, goats, pigs, and horses, causing an acute self-limiting gastroenteritis. Using the prototype species equine torovirus (EToV), we performed parallel RNA sequencing (RNA-seq) and ribosome profiling (Ribo-seq) to analyze the relative expression levels of the known torovirus proteins and transcripts, chimeric sequences produced via discontinuous RNA synthesis (a characteristic of the nidovirus replication cycle), and changes in host transcription and translation as a result of EToV infection. RNA sequencing confirmed that EToV utilizes a unique combination of discontinuous and nondiscontinuous RNA synthesis to produce its subgenomic RNAs (sgRNAs); indeed, we identified transcripts arising from both mechanisms that would result in sgRNAs encoding the nucleocapsid. Our ribosome profiling analysis revealed that ribosomes efficiently translate two novel CUG-initiated open reading frames (ORFs), located within the so-called 5′ untranslated region. We have termed the resulting proteins U1 and U2. Comparative genomic analysis confirmed that these ORFs are conserved across all available torovirus sequences, and the inferred amino acid sequences are subject to purifying selection, indicating that U1 and U2 are functionally relevant. This study provides the first high-resolution analysis of transcription and translation in this neglected group of livestock pathogens.

**IMPORTANCE** Toroviruses infect cattle, goats, pigs, and horses worldwide and can cause gastrointestinal disease. There is no treatment or vaccine, and their ability to spill over into humans has not been assessed. These viruses are related to important human pathogens, including severe acute respiratory syndrome (SARS) coronavirus, and they share some common features; however, the mechanism that they use to produce sgRNA molecules differs. Here, we performed deep sequencing to determine how equine torovirus produces sgRNAs. In doing so, we also identified two previously unknown open reading frames “hidden” within the genome. Together these results highlight the similarities and differences between this domestic animal virus and related pathogens of humans and livestock.

## Abstract (precis)

Toroviruses infect cattle, goats, pigs, and horses worldwide and can cause gastrointestinal disease. There is no treatment or vaccine, and their ability to spill over into humans has not been assessed. These viruses are related to important human pathogens, including severe acute respiratory syndrome (SARS) coronavirus, and they share some common features; however, the mechanism that they use to produce sgRNA molecules differs. Here, we performed deep sequencing to determine how equine torovirus produces sgRNAs. In doing so, we also identified two previously unknown open reading frames “hidden” within the genome. Together these results highlight the similarities and differences between this domestic animal virus and related pathogens of humans and livestock.

## INTRODUCTION

The order Nidovirales currently contains four families of positive-sense, single-stranded RNA viruses: the Coronaviridae, Arteriviridae, Roniviridae, and Mesoniviridae (1). Their grouping into the one taxonomic order is based upon replicase protein conservation, genome organization, and replication strategy. However, these viral families are nonetheless very diverse with respect to their virion structure, host range, pathogenic potential, and genome size.

The genus Torovirus (family Coronaviridae, subfamily Torovirinae) encompasses a range of species with worldwide distribution that infect domestic ungulates, including cattle, goats, sheep, pigs, and horses, causing an acute self-limiting gastroenteritis. Approximately 55% of cattle within the United Kingdom are seropositive for bovine torovirus, and this pathogen represents a significant burden to the industry (2, 3). Similarly, porcine torovirus is endemic to Europe and causes disease in production herds (4–6). Despite this, limited research has been conducted on these pathogens, and neither specific antiviral treatments nor vaccines are available. The prevalence of toroviruses in nondomestic reservoirs and potential for cross-species transmission have not been assessed, although they are known to undergo recombination events (7). The extensive research conducted upon the related coronaviruses would not necessarily be relevant in the event of an emerging torovirus infection due to the divergent nature of these viruses.

**FIG 1:**
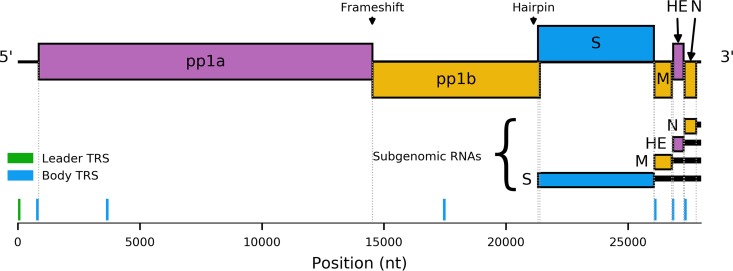
Schematic of the equine torovirus genome (EToV). Open reading frames (ORFs) are colored according to their respective reading frames (pink, phase 0; yellow, phase −1; blue, phase +1). Polyproteins pp1a and pp1ab are translated from genomic RNA, with pp1ab generated via −1 programmed ribosomal frameshifting. Structural proteins are translated from a series of sgRNAs. Untranslated regions of sgRNAs are represented by black bars. The leader transcription regulatory sequence (TRS) (green) and putative body TRSs (blue) are displayed below the viral genome. The frameshift site and a putative RNA hairpin involved in S sgRNA synthesis are indicated above the genome.

The genomes of members of the order Nidovirales are positive-sense, polycistronic RNAs. One of the hallmarks of this virus order is the utilization of an unusual transcription mechanism to express the genes encoding structural and accessory proteins, which reside downstream of the large replicase open reading frames (ORFs) 1a and 1b (Fig. 1). These proteins are typically translated from a nested set of 3′-coterminal subgenomic mRNAs (sg mRNAs). Although, with the exception of the smallest species, these sgRNAs are structurally polycistronic, translation is normally limited to the 5′ ORF of each mRNA. Studies of coronaviruses and arteriviruses have revealed that they produce negative-sense subgenome-sized RNAs via a mechanism of discontinuous extension (8) (recently reviewed by Sola et al. [9]). This process may resemble homology-assisted copy-choice recombination (10) and requires the presence of multiple copies of a species-specific short motif, the transcription regulatory sequence (TRS). TRS motifs are located immediately upstream of the structural protein-coding ORFs (body TRSs) and within the 5′-untranslated region (UTR; leader TRS).

Negative-strand RNA synthesis initiates at the 3′ end of the positive-sense viral genome. When the RNA-dependent RNA polymerase (RdRp) has copied a TRS sequence, a template-switching event may occur during which the anti-TRS at the 3′ end of the nascent strand base pairs with the leader TRS within the 5′ UTR. Transcription reinitiates and continues to the 5′ end of the genomic template. The resulting “anti-leader” sequence that is added ranges from 55 to 92 nucleotides (nt) in coronaviruses to ∼200 nt in arteriviruses. These negative-sense transcripts are therefore 5′- and 3′-coterminal with the full-length negative RNA strand and are identifiable as chimeras with distinct flanking sequences adjacent to the core TRS. The anti-leader sequence in each of the negative-sense templates then functions as a promoter to drive synthesis of a mirror set of positive-sense sgRNAs that are translated to produce the structural proteins.

However, not all details of the mechanism outlined above are wholly conserved across the Nidovirales (11). Specifically, the two sg mRNAs of roniviruses (pathogens of shrimp) do not possess conserved 5′ leader sequences, indicative of the lack of a discontinuous step during their production (12). Despite the presence of a conserved body TRS in each subgenomic mRNA, an equivalent leader TRS is not readily identifiable in the 5′ UTR. It may therefore be reasoned that the ronivirus body TRSs stimulate termination of negative-strand RNA synthesis without a subsequent RdRp template switch and reinitiation. Mesoniviruses (a branch of Nidovirales recently identified in insects) are thought to produce two major sgRNAs possessing leader sequences of different lengths, indicating that the nidoviral mechanism for discontinuous RNA synthesis can allow two very different leader/body TRS pairs to be utilized in a single viral species (13).

Toroviruses appear to represent a nidovirus subgroup with a remarkably flexible transcription strategy: equine torovirus (EToV) possesses a leader TRS-like sequence (CUUUAGA), but it is only involved in the synthesis of the mRNA used for expression of the spike (S) protein gene (14). Despite similarities to the corona- and arteriviral mechanism, the preceding leader sequence incorporated into this mRNA is merely 6 nt in length (ACGUAU). Additionally, this case is unusual in that the template switch event is thought to be prompted by an RNA structure, a predicted RNA hairpin upstream of the S protein gene, rather than a body TRS (14). Conventional body TRSs are located upstream of the three remaining structural protein genes, yet a nondiscontinuous mechanism is utilized for the production of the corresponding sgRNAs, as is the case for roniviruses. As a result, the sg mRNAs for membrane (M), nucleocapsid (N), and hemagglutinin-esterase (HE) do not normally possess a common 5′ leader sequence; they each possess a variable and unique extended version of the TRS at their 5′ end. It is clear there are significant differences between the various Nidovirales families in how they synthesize their sgRNAs.

Here, we describe the first high-resolution analysis of viral transcription during infection by EToV, which is one of the few toroviruses that can be propagated in cell culture (15, 16). RNA sequencing (RNA-seq) confirmed previous reports that EToV utilizes a unique combination of both discontinuous and nondiscontinuous RNA synthesis to generate its repertoire of sgRNAs. Strikingly, we also identified a small proportion of chimeric transcripts spanning from the leader to the body TRS of the N protein gene, indicating that discontinuous and nondiscontinuous mechanisms compete in this location. We also identified numerous locations across the genome where noncanonical RdRp template-switching occurs, leading to a vast array of (presumably mostly defective or nonfunctional) chimeric transcripts.

Ribosome profiling (Ribo-seq) conducted in tandem with the RNA-seq indicated that ribosomes actively translate within the so-called 5′ UTR. Further analysis confirmed the existence of two novel ORFs in this region, which are conserved in all torovirus genome sequences analyzed to date. The specific function(s) of these proteins will be the topic of future work. Together, these results provide an overview of the transcriptional and translational events that accompany infection by this wide-ranging pathogen.

## RESULTS

### Tandem RNA-seq and Ribo-seq of EToV-infected cells.

We conducted tandem RNA-seq and Ribo-seq of EToV-infected equine dermal (ED) cells. Two biological replicates of virus-infected and mock-infected cells were analyzed, generating 25 to 53 million reads per sample. For RNA-seq, 77 to 92% of reads mapped to the host genome, of which a mean of 1.5% mapped to rRNA, 19% to mRNA, 32% to noncoding RNA (ncRNA), and 47% elsewhere in the genome. For Ribo-seq, 46 to 60% of reads mapped to the host genome, of which a mean of 56% mapped to rRNA, 13% to mRNA, 4.9% to ncRNA, and 26% elsewhere in the genome (see Table S1 in the supplemental material). Totals of 1.3% and 2.3% of reads mapped to the virus genome in the two EToV-infected RNA-seq replicates and 0.41% and 0.21% in the two EToV-infected Ribo-seq replicates.

The viral genome was assembled *de novo* from RNA-seq reads and confirmed as EToV, Berne isolate. A single 27,694-nt contig was assembled representing almost the entire viral genome. Only 18 nt at the 5′ terminus and 300 nt at the 3′ terminus failed to assemble automatically; however, these regions were clearly covered by reads consistent with the reference sequence on inspection and so were added manually to the consensus sequence. Four single-nucleotide changes were present in all reads, but not the reference sequence compiled from previous sequencing data, at positions 18078 (ORF 1b, C>U), 21429 (ORF S, A>U), 21814 (ORF S, C>A), and 25596 (ORF S, C>U).

**FIG 2:**
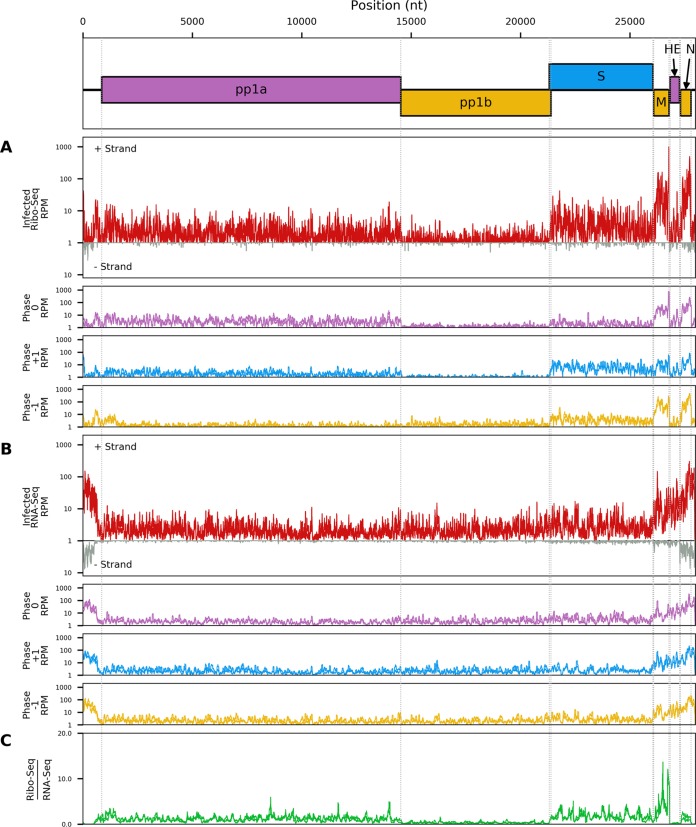
Read density of Ribo-seq (A) and RNA-seq (B) reads across the viral genome from EToV-infected cells. Red lines represent total reads per million mapped reads at each position; pink, reads in phase 0; yellow, phase −1; blue, phase +1. Densities are smoothed with a 15-nt running mean filter and plotted on a log_10_(1 + *x*) scale. Negative-sense reads (gray) are displayed below the *x* axis for total reads only. Each line represents a single replicate. For Ribo-seq reads, a +12-nt offset has been applied to read 5′-end positions to map approximate P-site positions. (C) The positive-sense Ribo-seq/RNA-seq ratio after applying a 100-nt running mean filter to each distribution. Each line represents one of the two paired Ribo-seq and RNA-seq replicates.

**FIG 3:**
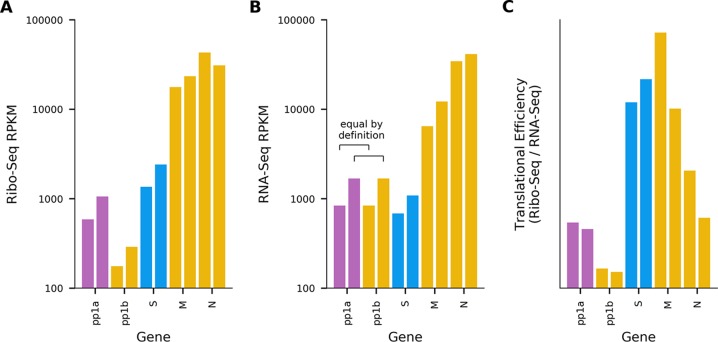
Relative gene expression levels. (A) Ribo-seq density, in reads per kilobase per million mapped reads (RPKM), for each ORF in the EToV genome. For each ORF, only reads mapping in the predominant phase (i.e., mapping to first positions of codons) were included. (B) Decumulated RNA-seq density in RPKM for each ORF. For sgRNAs, density was calculated across the regions used for Ribo-seq in panel A; for genomic RNAs the regions for ORF1a and ORF1b were combined, as these ORFs are both translated from gRNA. A decumulation strategy was used to correct for the fact that the measured RNA density in 3′ ORFs derives from multiple 3′-coterminal transcripts (see Materials and Methods). (C) Translation efficiency for each gene in the EToV genome, calculated as Ribo-seq density/decumulated RNA-seq density. For each ORF, the two bars represent two repeats.

The distribution of reads on the virus genome and the phasing of these reads are shown in Fig. 2. There was good coverage across the viral genome for both RNA-seq and Ribo-seq. The Ribo-seq/RNA-seq ratio along the genome was calculated (Fig. 2C) to estimate translation efficiency (note that this simple estimate is naïve, since it does not account for the fact that the genomic RNA and different sgRNA species overlap one another). Ribo-seq density, RNA-seq density, and translational efficiency were also calculated separately for each ORF (Fig. 3), based on the density of Ribo-seq reads in each ORF divided by the density of the RNA-seq reads for either the same region (for sgRNAs) or the region of the genome which does not overlap the sgRNAs (for genomic RNA [gRNA]). RNA-seq density was adjusted based on the “decumulation” methodology described previously (17) (see Materials and Methods) to account for the fact that not all of the RNA-seq density in the 3′ ORFs derives from transcripts from which the ORFs can be expressed. Ribo-seq coverage is much higher toward the 3′ end of the genome, particularly across the M and N genes, reflecting the translation of abundant sgRNAs in this region (Fig. 2 and 3). ORFs 1a and 1b contain a considerably lower density of Ribo-seq reads. The relatively low translation efficiencies calculated for ORFs 1a and 1b may be partly due to some gRNA being packaged (or destined for packaging) and unavailable for translation but still contributing to the estimate of gRNA RNA-seq density. They may also represent regulation of expression to control the amount of the replicase proteins, which are generally required at a lower level than structural proteins. ORF1a has a higher Ribo-seq density and a higher translational efficiency than ORF1b, reflecting the proportion of ribosomes terminating at the ORF1a stop codon and not undergoing the −1 frameshift into ORF1b (Fig. 2 and 3). As expected, RNA-seq density is similar across ORF1a and ORF1b, as both are present only on the full-length genomic RNA (Fig. 2). The region covering the HE ORF also has low ribosomal coverage (Fig. 2), which may be due to the fact that, in contrast to other toroviruses, the EToV HE gene is nonfunctional due to a large deletion, including the canonical AUG (18). HE is not shown in Fig. 3, as the HE transcript is much less abundant than the upstream M transcript, which makes the decumulation procedure susceptible to noise (17). Translational efficiency appears highest for the M and S sgRNAs. The high RNA-seq density in the 5′ UTR may be indicative of one or more defective interfering (DI) RNAs in the sample (see below). Ribosome-protected fragments (RPFs) were also identified mapping to the second half of the 5′ UTR, mostly in the +2/−1 frame with respect to ORF1a (Fig. 2A).

**FIG 4:**
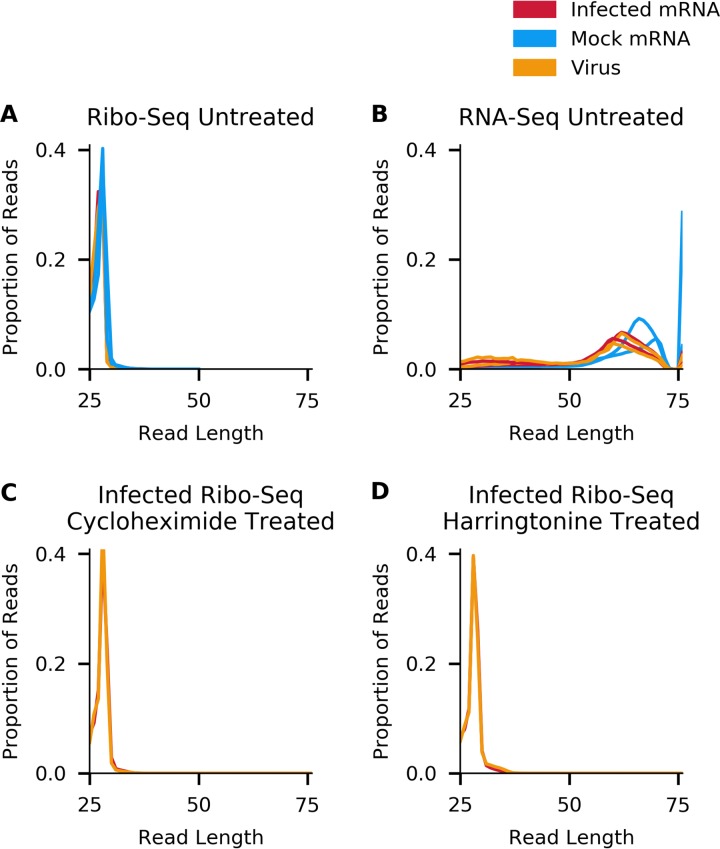
Comparison of read length distributions for reads mapping to EToV in infected cells (orange), host mRNAs in noninfected cells (blue), and host mRNAs in infected cells (red) for Ribo-seq data in non-drug-treated cells (A), RNA-seq data in non-drug-treated cells (B), Ribo-seq data in cycloheximide-treated cells (C), and Ribo-seq data in harringtonine-treated cells (D).

**FIG 5:**
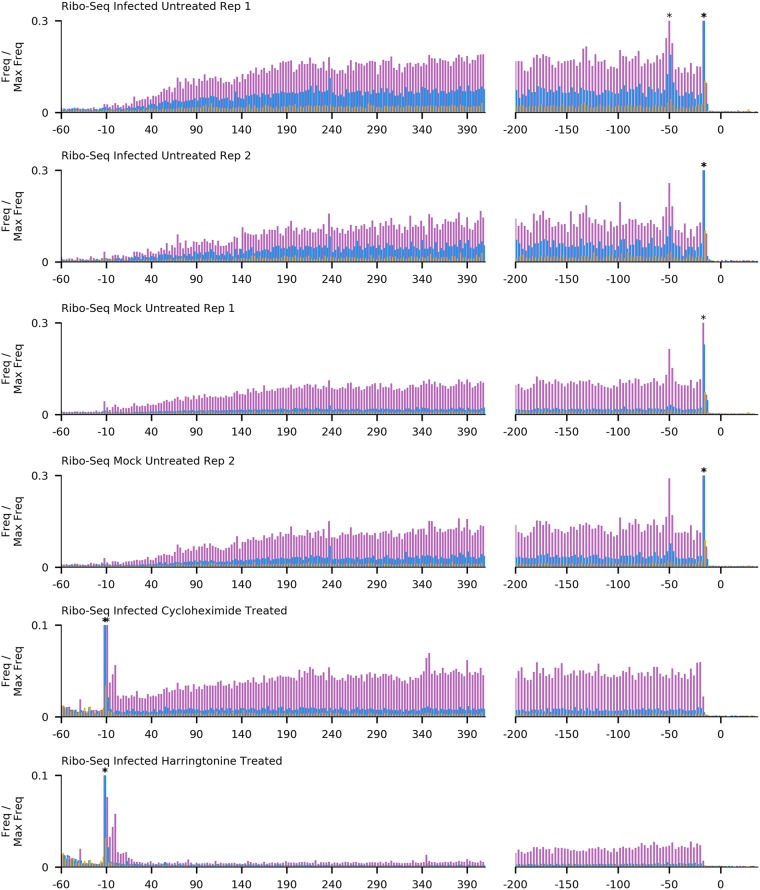
Histograms of Ribo-seq read 5′-end positions (in nucleotides) relative to annotated initiation (left) and termination (right) sites, summed across all host mRNAs. Bars are colored by phase relative to the first base of the start codon (pink, phase 0; blue, phase +1; yellow, phase −1). Histograms are scaled so that the maximum value is 1. For clarity, the *y* axis is cropped at 0.3 for non-drug-treated and 0.1 for drug-treated cells; bars which extended beyond this point are marked with an asterisk.

**FIG 6:**
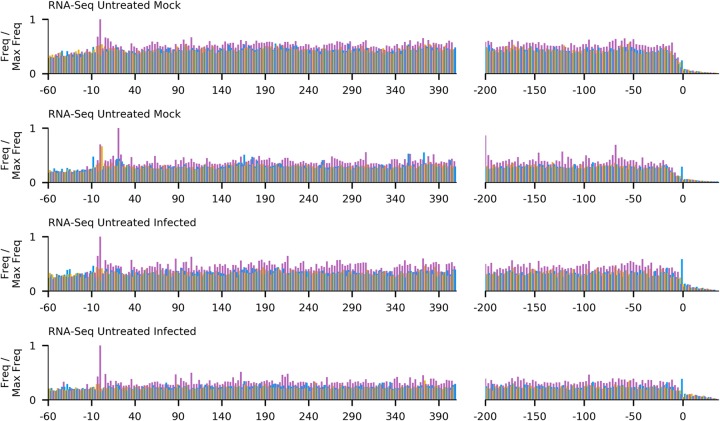
Histograms of RNA-seq read 5′-end positions (in nucleotides) relative to annotated initiation (left) and termination (right) sites, summed across all host mRNAs. Bars are colored by phase relative to the first base of the start codon (pink, phase 0; blue, phase +1; yellow, phase −1). Histograms are scaled so that the maximum value is 1.

**FIG 7:**
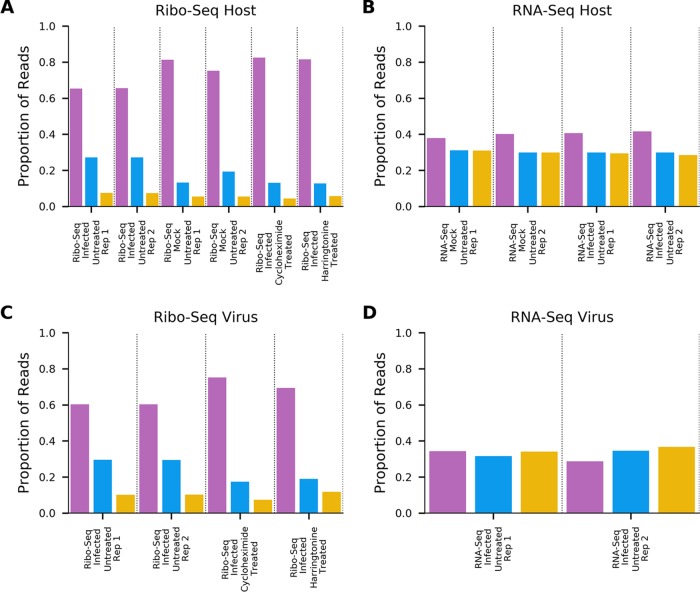
Phasing of the 5′ ends of reads (pink, phase 0; blue, phase +1; yellow, phase −1) for Ribo-seq reads mapping to host mRNA coding regions (A), RNA-seq reads mapping to host mRNA coding regions (B), Ribo-seq reads mapping to virus mRNA coding regions (C), and RNA-seq reads mapping to virus mRNA coding regions (D).

To calculate the length distributions of host- and virus-mapped RPFs, we used reads mapping within coding regions. After adaptor trimming, the majority (75%) of Ribo-seq reads were 27 to 29 nt in length, which is consistent with the expected size of mammalian ribosome footprints. As expected, the distribution of read lengths for RNA-seq was much broader, peaking between 60 and 70 nt (Fig. 4). For quality control, histograms of the 5′ end positions of host mRNA Ribo-seq and RNA-seq reads relative to initiation and termination codons were constructed (Fig. 5 and 6). This confirmed we had high-quality RPFs arising from host transcripts, with strong triplet periodicity (phasing) and very few reads mapping to 3′ UTRs. As in other data sets, a ramp effect of decreased RPF density was seen over a region of ∼30 codons following initiation sites; however, unusually, in this data set we did not observe a density peak at the initiation site itself (see Irigoyen et al. [17]). This may be due to the flash freezing without cycloheximide pretreatment used for these samples, as for a later cycloheximide-treated sample this peak is present (Fig. 5). Within coding sequences, the 5′ ends of the majority of reads from the host (65 to 81%) and virus (60 to 75%) mapped to the first codon positions (Fig. 7).

The relative RPF density allowed us to estimate the efficiency of ribosomal frameshifting in the context of virus infection. After translating ORF1a, a proportion of ribosomes undergo a −1 ribosomal frameshift to translate ORF1b (19). This is (presumably) required to produce a specific ratio of pp1a to pp1ab, thereby controlling the ratio of RNA-synthesizing enzymes, such as RdRp and helicase, to other components of the replicase complex, including the proteinases and transmembrane subunits in ORF1a. The ORF1a/1b −1 ribosomal frameshifting event is stimulated by a pseudoknot structure 3′ adjacent to the U_UUA_AAC slippery heptanucleotide frameshift site. The efficiency of −1 ribosomal frameshifting (measured by dividing the mean RPF density in ORF1b by the mean density in ORF1a) was estimated to be 29.9% for replicate 1 and 27.5% for replicate 2, which is in accordance with the rates measured previously outside the context of virus infection (20 to 30%) (19).

### RNA sequencing indicates both discontinuous and nondiscontinuous mechanisms are utilized for N protein gene sgRNA synthesis.

**FIG 8:**
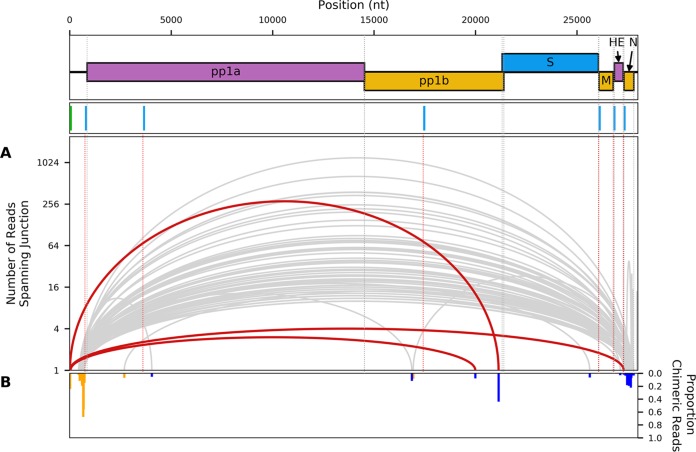
Analysis of chimeric viral reads. (A) Sashimi plot showing junctions in the EToV genome, across which chimeric RNA-seq reads were identified in EToV-infected, non-drug-treated samples. Chimeric reads were defined as reads for which the intact read could not be mapped but for which the 5′ and 3′ ends could be uniquely mapped to noncontiguous regions of the EToV genome. Junctions that were covered by at least 10 chimeric reads (gray) and/or for which the 5′ section of the read contained the full 5′ leader sequence and leader TRS (red) were identified and adjacent positions merged. These junctions are shown as curved lines connecting the position of the 3′ end of the 5′-mapped segment of the read and the 5′ end of the 3′-mapped segment of the read. The apical height of each curved line shows the absolute number of reads spanning this junction on a log_10_(1 + *x*) scale. (B) Inverted bar chart showing, for the 5′ (orange) and 3′ (blue) breakpoints for each junction, the number of chimeric reads as a fraction of the total number of chimeric and nonchimeric reads at each site.

RNA sequencing reads that did not map to either the viral genome or host databases were analyzed for containing potential viral chimeric junctions, indicative of leader-to-body joining during discontinuous sgRNA synthesis (Fig. 8). Relative abundances were calculated by normalizing read counts to the number of nonchimeric reads spanning each junction. Between the two replicates combined, 8,330 reads were identified as chimeras, mapping to 2,837 putative junction sites. Of these, 213 were considered to be highly supported by the data, either due to being identified in at least 10 chimeric reads or containing the full 5′ leader and TRS sequence. Adjacent donor or acceptor sites were then merged (see Materials and Methods), leaving 70 unique junctions (Fig. 8).

Three chimeric junctions were identified where the first nucleotide of the corresponding read mapped to the first nucleotide of the viral genome. Of these, one junction was consistent with the previously characterized sgRNA produced via discontinuous RNA synthesis encoding the S gene (280 reads, or 3% of total chimeric reads) (14). These reads spanned the entire leader-body junction of the S gene, possessing 14 to 18 nt of the 5′ UTR (i.e., the actual 5′-derived sequence is at least 14 nt, ACGUAUCUUUAGAA, comprising the so-called 6-nt leader, the leader TRS CUUUAGA, and an additional A), followed by the stretch of ORF1b just upstream of the S gene. A second set of transcripts containing 5′ leader sequence was identified by four unique reads starting with the 5′ leader (ACGUAU) and TRS sequence (CUUUAGA), where the remainder of the read mapped to the start of the N gene. This indicates that, contrary to previous reports, low levels of discontinuous RNA synthesis are used during production of the N gene negative-strand RNA. The final chimaera, which included the 6-nt leader, was represented by three reads. These reads included 44 to 46 nt of the 5′ UTR (i.e., significantly more than the normal leader TRS) followed by a sequence mapping to positions 19,987 to 19,989, which is within ORF1b.

A substantial number of additional chimeric reads were identified, indicative of non-TRS-driven cases of discontinuous RNA synthesis, although formally it is possible that some of these are template-switching artifacts introduced during library preparation and/or sequencing. Additionally, a large number of reads spanning from the 5′ UTR to either within the N protein gene or the 3′ UTR were identified. Indeed, the only junction represented by over 1,000 reads spanned nucleotides 673 to 27649; similarly, the second most commonly identified junction, with 642 reads, spanned 687 to 27550. If chimeric reads were predominantly a sequencing artifact, the abundance of any particular chimaera would be approximately proportional to the product of the abundances of the sequences from which the 5′ and 3′ ends of the chimera are derived (with some variation due to sequence-specific biases), thus a high density of chimeras would be expected to fall entirely within the N transcript. In contrast, most of the observed chimeric reads were between N and the 5′ UTR. The relative paucity of reads mapping to generic locations in the ORF1ab region also argues against the majority of chimeras being simply artifactual. The 5′ UTR preference may be due to genome circularization during negative-sense synthesis, as has been proposed for coronaviruses (20). Alternatively, these may derive from autonomously replicating defective interfering RNAs rather than multiple independent RNA translocation and reinitiation events. Such defective interfering RNAs have been extensively analyzed previously and are a common complication of EToV studies relying on high-multiplicity-of-infection (MOI) infections in cell culture (21). Consistent with the high level of 5′ UTR:N chimeric sequences, there was high RNA-seq density throughout much of the 5′ UTR, with the 3′ extent of the region of high density coinciding approximately with the region to which a large number of the chimeric 5′ ends mapped (Fig. 2 and 8).

### Gene expression analysis indicates multiple pathways are perturbed by EToV infection.

**FIG 9:**
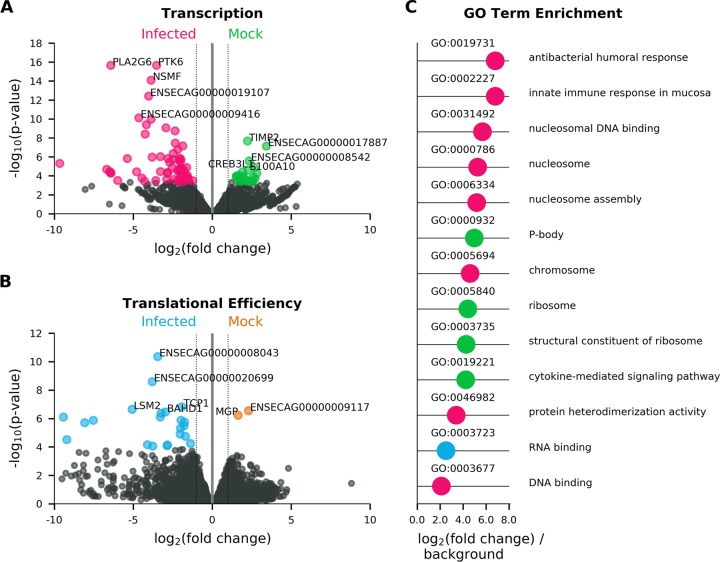
Volcano plots showing the results of differential transcription analysis performed using DESeq2 (43) (A) and differential translation efficiency analysis performed using Xtail (B) between cells infected with EToV (infected) and uninfected cells (mock). Genes which were expressed at significantly higher levels (FDR of ≤0.05 and absolute log_2_ fold change of ≥1) in infected cells are highlighted in pink (transcription) (A) and blue (translational efficiency) (B). Genes which were expressed at significantly higher levels in mock-infected cells are highlighted in green (transcription) (A) and orange (translational efficiency) (B). The five most significant genes in each category are labeled with the gene symbol, where available, and otherwise with the Ensembl gene identifier. (C) Absolute log_2_ fold change for all gene ontology (GO) terms which were significantly overrepresented compared to a background of all horse protein-coding genes for genes significantly more transcribed in infected cells (pink), genes significantly more efficiently translated in infected cells (blue), and genes significantly more transcribed in mock cells (green). No terms were identified for genes significantly more efficiently translated in mock cells.

The RNA-seq data were analyzed to identify genes that were differentially expressed between virus-infected and mock-infected ED cells. We identified 61 genes that were upregulated in virus-infected cells, among which eight gene ontology (GO) terms were overrepresented, mostly related to the nucleosome or immune responses (Fig. 9). We found 24 genes that were downregulated in infected cells, among which four GO terms were overrepresented, two of which were related to the ribosome. We also analyzed differential translational efficiency (based on the RPF/mRNA ratio) between mock- and virus-infected cells. We identified 22 genes that were translated more efficiently in infected cells; GO analysis indicated that these genes tend to encode proteins that are involved in RNA binding. Only two genes were found to be translated less efficiently in infected cells than mock-infected cells (Fig. 9 and Table S2). Note that these analyses measure changes in individual genes relative to the global mean and do not inform on global changes in host transcription or translation as a result of virus infection.

### Two additional proteins are translated from 5′ CUG-initiated ORFs.

**FIG 10:**
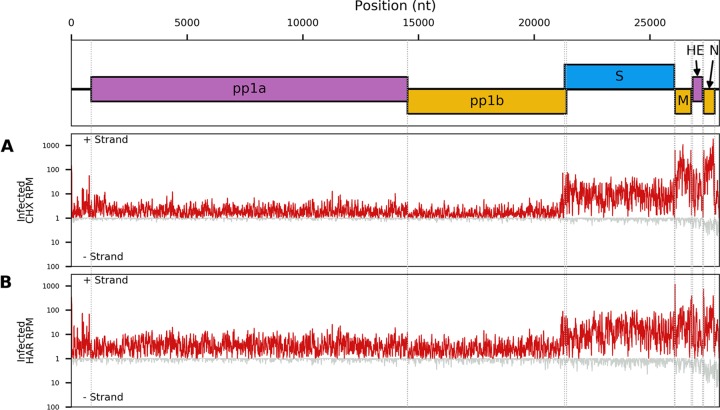
Read density of Ribo-seq reads along the viral genome for EToV-infected cells pretreated with cycloheximide (A) or harringtonine (B). Red lines represent total reads per million mapped reads (RPM) at each position. Densities are smoothed with a 15-nt running mean filter and plotted on a log_10_(1 + *x*) scale. Negative-sense reads (gray) are displayed below the *x* axis. A +12-nt offset has been applied to read 5′-end positions to map approximate P-site positions.

Our initial data set indicated an excess density of ribosomes translating within the +2/−1 frame upstream of ORF1a and overlapping the 5′ end of ORF1a (Fig. 2A). To further investigate this, we repeated the ribosome profiling using infected cells treated with translation inhibitors prior to flash freezing (harringtonine, or HAR, and/or cycloheximide, or CHX). HAR specifically arrests initiating ribosomes while allowing runoff of elongating ribosomes; conversely, CHX stalls elongating ribosomes while allowing ongoing accumulation at initiation sites. Our quality control analysis confirmed the data sets were of similar quality to our previous experiment (Fig. 4, 5, and 7), and mapping of the RPFs provided good coverage of the EToV genome (Fig. 10).

**FIG 11:**
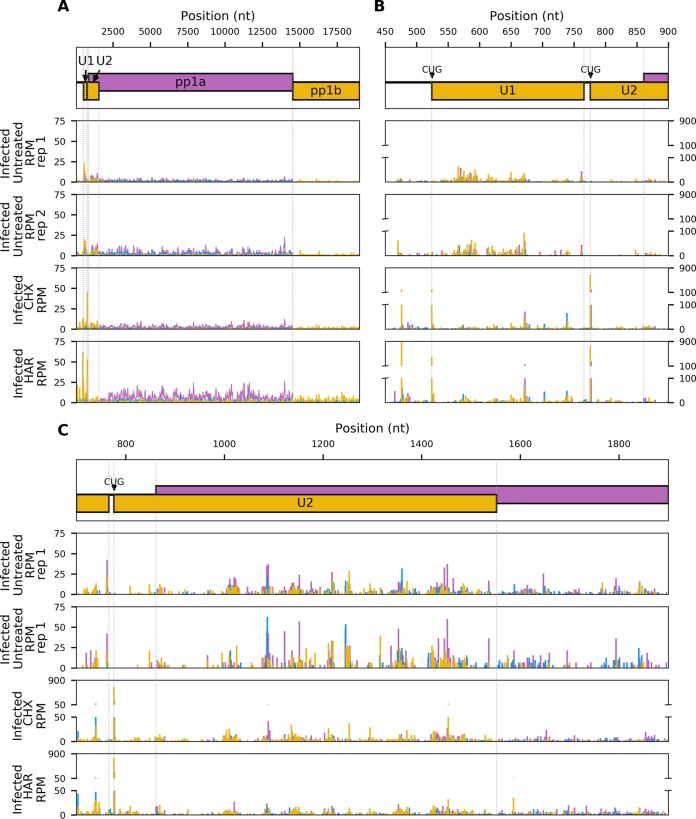
Read density of Ribo-seq reads across U1, U2, and ORF1a (A), the U1 ORF and surrounding regions (B), and the U2 ORF and surrounding regions (C) for EToV-infected cells with no drug treatment or with cycloheximide or harringtonine pretreatment. Pink, reads in phase 0; yellow, phase −1; blue, phase +1. Graphs show total reads per million mapped reads (RPM) at each position. In panel A densities are smoothed with a 15-nt running mean filter, while panels B and C show the RPM counts at single-nucleotide resolution. Each plot represents a single replicate. A +12-nt offset has been applied to read 5′-end positions to map approximate P-site positions.

This Ribo-seq data confirmed translation of two ORFs located within the so-called 5′ UTR and overlapping the 5′ end of ORF1a. We have termed these U1 (80 codons) and U2 (258 codons). We predict that translation of both U1 and U2 is initiated from CUG codons, as a close inspection indicated that ribosomes accumulate at these two sites (Fig. 11). It must be noted that pretreatment with CHX or HAR can introduce artifacts into ribosome profiling data: CHX can lead to an excess of RPF density over ∼30 codons following initiation sites when cells are stressed (17, 22). It has also been suggested that both drugs can promote upstream initiation due to scanning preinitiation complexes stacking behind ribosomes paused at canonical initiation sites (23). However, the distance between the U1 CUG, the U2 CUG, and the ORF1a initiation site, besides observation of efficient translation of U2 downstream of the ORF1a initiation site (Fig. 11A), makes these artifacts unlikely to be significant confounding factors in the case of U1 and U2.

**FIG 12:**
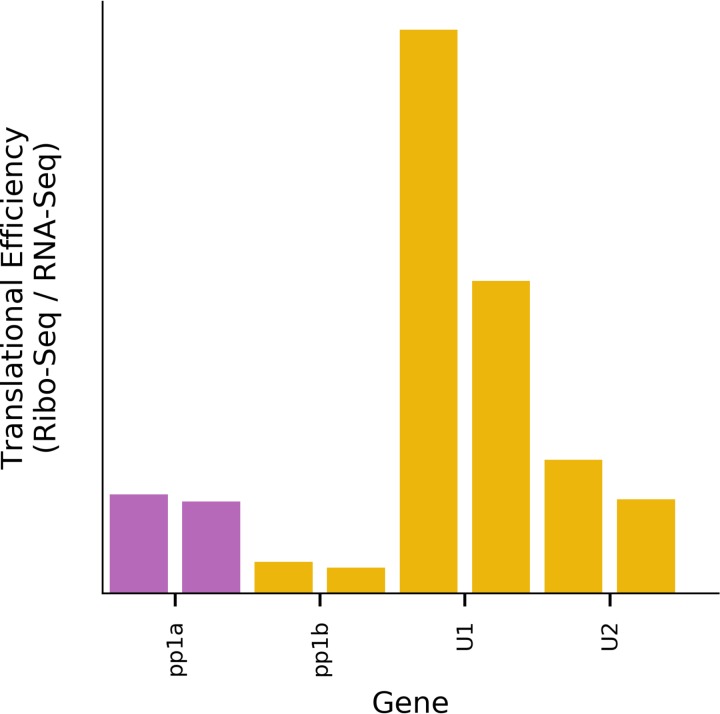
Relative translation efficiencies for U1, U2, ORF1a, and ORF1b. To reduce misassignment of reads in the U2/ORF1a overlap region, for all ORFs only reads mapping in the predominant phase (i.e., mapping to first positions of codons) were included. Ribo-seq densities were divided by the ORF1ab RNA-seq densities for the corresponding paired sample. For each ORF, the two bars represent two repeats.

Revisiting our first nondrug-treated data set, we calculated the RPF densities and translational efficiencies within the U1 and U2 ORFs (Fig. 12). U1 has a higher translational efficiency than any of the other ORFs translated from the genomic RNA, whereas U2 has a translational efficiency similar to that of ORF1a.

**FIG 13:**
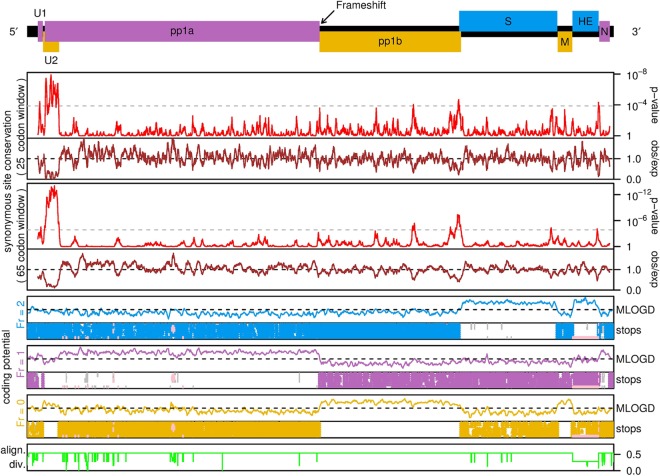
Coding potential statistics for the torovirus genome. A map of the torovirus genome is shown at the top. Breda virus (AY427798.1) was used as the reference genome for this analysis, since EToV has a deletion in the HE gene. In Breda virus, U1 is in frame with ORF1a due to a 2-nt insertion relative to EToV in the short noncoding region between U1 and U2. The next four panels show an analysis of synonymous site conservation in the concatenated coding ORFs (with the reading frame of the longer ORF being used wherever two ORFs overlap). Red lines show the probability that the degree of conservation within a given window (25 or 65 codons, as indicated) could be obtained under a null model of neutral evolution at synonymous sites, whereas brown lines depict the absolute amount of conservation, as represented by the ratio of the observed number of substitutions within a given window to the number expected under the null model (obs/exp). Greatly enhanced synonymous site conservation is seen in the region of ORF1a that is overlapped by the U2 ORF. The next three panels show MLOGD coding potential scores and stop codon plots for each of the three reading frames. The positions of stop codons are shown for each of the eight torovirus sequences mapped onto the Breda virus reference sequence coordinates. Note the conserved absence of stop codons in the U1 and U2 ORFs. MLOGD was applied in a 40-codon sliding window (5-codon step size). Positive scores indicate that the sequence is likely to be coding in the given reading frame. Note the positive scores within the U1 and U2 ORFs besides the previously known ORFs. The bottom panel (green line) indicates the total amount of phylogenetic divergence contributing to the analyses at each alignment position (regions containing alignment gaps have reduced summed divergence, leading to reduced statistical power). Pink regions in the stop codon plots (e.g., EToV sequence in the HE region) indicate regions excluded from the analyses due to poor or locally out-of-frame mapping to the Breda reference sequence (see Firth for details [31]).

To assess the coding potential of U1, we calculated the ratio of nonsynonymous to synonymous substitutions (*dN*/*dS*), where *dN*/*dS* of <1 indicates selection against nonsynonymous substitutions, which is a strong indicator that a sequence encodes a functional protein. Application of codeml (24) to a codon alignment of eight torovirus U1 nucleotide sequences resulted in a *dN*/*dS* estimate of 0.31 ± 0.08, indicating that the U1 ORF encodes a functional protein. MLOGD (25) uses a principle similar to the *dN*/*dS* statistic but also accounts for conservative amino acid substitutions (i.e., similar physicochemical properties), being more probable than nonconservative substitutions in biologically functional polypeptides. MLOGD 3-frame “sliding window” analysis of a full-genome alignment revealed a strong coding signature in the known protein-coding ORFs (as expected) and also in the U1 ORF (Fig. 13).

We previously predicted the existence of U2 via an analysis of coding potential and synonymous site conservation across the two torovirus genomes available at that time (26). Six additional torovirus genome sequences have now become available. We therefore extended the bioinformatics analysis using all eight currently available torovirus genome sequences (Fig. 13). Since the U2 ORF overlaps ORF1a, leading to constraint on *dS*, the *dN*/*dS* analysis is not appropriate for U2. MLOGD analysis indicated that the U2 ORF has a higher coding potential than the corresponding part of ORF1a (Fig. 13). Overlapping genes are thought mainly to evolve through “overprinting” of an ancestral gene by the *de novo* gene (27). The *de novo* gene product is often an accessory protein and often disordered (28). Interestingly, the fragment of pp1a encoded by the region of ORF1a that is overlapped by U2 has no tblastn (29) nor HHpred (30) homologues outside the Torovirus genus. Thus, it is unclear which of U2 and the N-terminal domain of pp1a is ancestral. To provide further comparative genomic evidence for the functionality of U2, we used synplot2 to assess conservation at synonymous sites in the ORF1a reading frame, since overlapping functional elements are expected to place extra constraints on synonymous site evolution (31). Consistent with the earlier 2-sequence analysis (26), synplot2 revealed greatly enhanced ORF1a-frame synonymous-site conservation in a region coinciding precisely with the conserved absence of stop codons that defines the U2 ORF (Fig. 13), with the mean rate of synonymous substitutions in that region being 0.20 of the genome average. Summed over the 230-codon overlap region, the probability (*P* value) that the observed level of conservation would occur by chance is 6.5 × 10^−40^.

**FIG 14:**
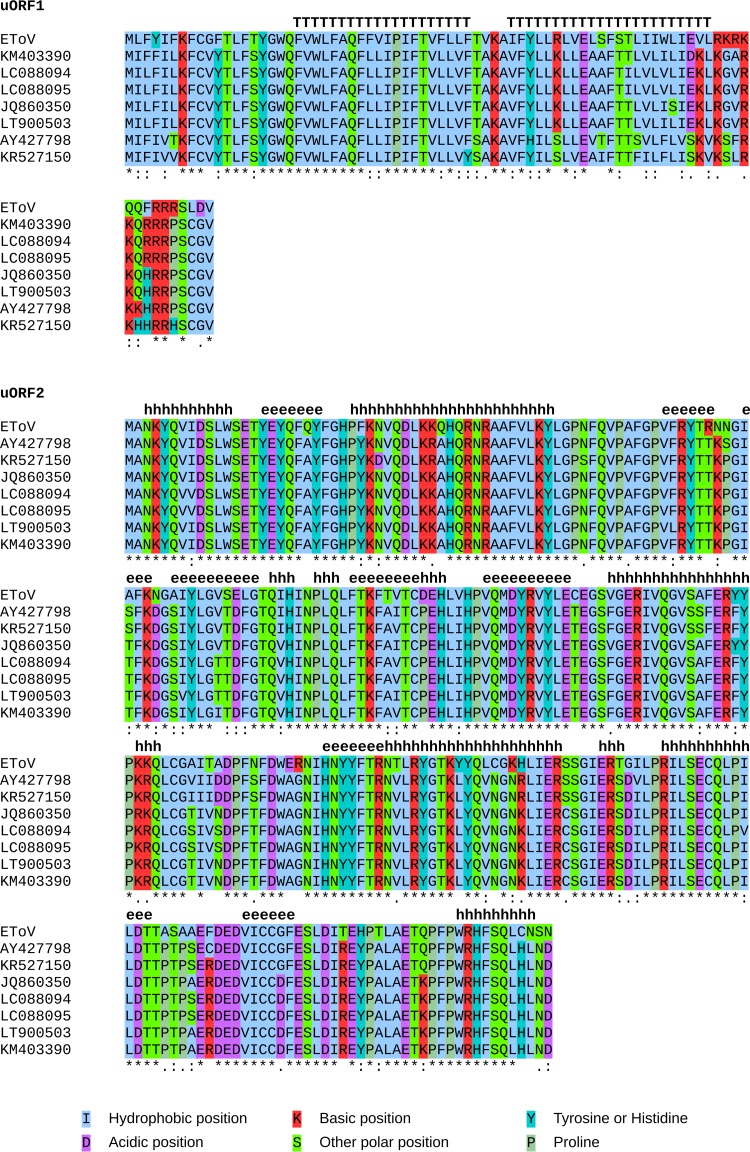
Conservation of uORF1 and uORF2 in the eight publicly available torovirus genomes. Individual amino acid residues are colored according to their biochemical properties. Asterisks below the alignments indicate conservation, and predicted secondary structures are annotated above the alignments (h, helix; e, beta strand; T, transmembrane).

Both U1 and U2 are conserved in all eight torovirus sequences, with no variation in length or initiation or termination position (Fig. 14). In all sequences, U1 and U2 begin with a CUG codon in a strong initiation context (A at −3 for U1 and A at −3 and G at +4 for U2) (32). The U1 protein is predicted to contain two central transmembrane domains and has a C terminus containing many charged amino acids. The U2 protein is predicted to form alternating α helix and antiparallel β sheet domains; however, no structural homologs were found through searches of public databases (33–35). Their function(s) will be the topic of future work.

## DISCUSSION

### RNA-seq reveals the complexity of torovirus transcription mechanisms.

**FIG 15:**
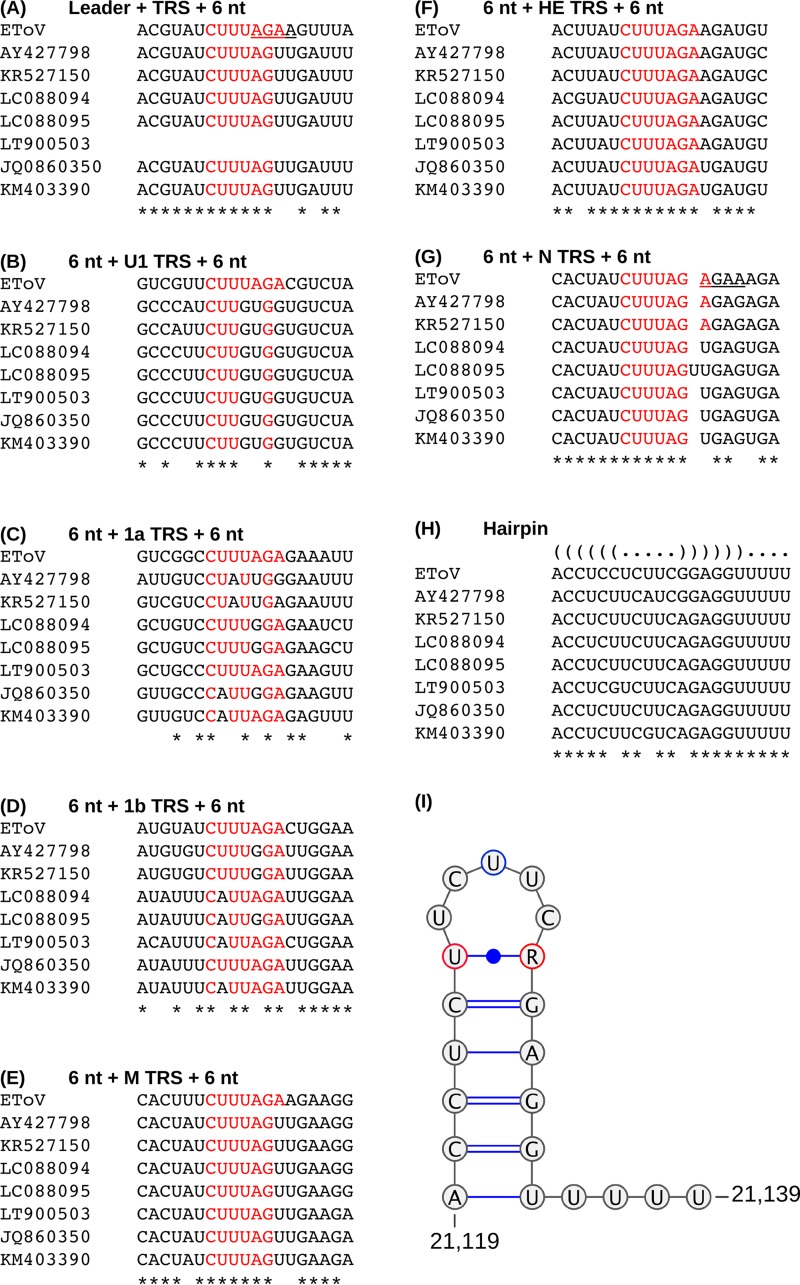
Conservation of TRSs and regulatory structures in the eight publicly available torovirus genomes. Regions were selected based on the presence of a putative TRS in the EToV genome. The TRS and six flanking nucleotides are displayed; putative TRS nucleotides are highlighted in red. Nucleotide conservation between all eight sequences is indicated by an asterisk. The predicted hairpin structure (I) is based upon nucleotide conservation across all eight genomes. Variant nucleotides are circled in either red (covariance indicates the predicted pairing may occur in all but one genome) or blue (variable). R indicates a purine exists in all genomes.

The factors influencing which transcriptional mechanism is utilized for the synthesis of each sgRNA during torovirus replication have not been fully elucidated. The EToV genome contains seven occurrences of the canonical TRS motif (CUUUAGA): within the 5′ UTR (leader TRS), the end of U1, central ORF1a, central ORF1b, and immediately before the M, HE, and N ORFs (Fig. 1). Consistent with experimental evidence (14), we did not identify any chimeric transcripts encompassing the body TRS of M or HE or those within ORF1b or ORF1a. It appears that these sites do not stimulate template switching during negative-strand RNA synthesis. The nucleotides flanking the N, M, and HE TRSs are semiconserved (Fig. 15), and it has been suggested previously that the motif definition should be extended to cACN_3–4_CUUUAGA to reflect this (36). It is likely that these flanking nucleotides contribute to the degree of utilization.

For the S gene, our results lend support to the hypothesis suggested previously that a short conserved RNA hairpin, 174 nt upstream of the initiation codon, is involved in stimulating template switching for this sgRNA (14) (Fig. 15). The chimeric junction occurs within the run of uridines 3′-adjacent to the hairpin (Fig. 15I), indicating that template switching is prompted by the RdRp encountering this structure. This is in contrast to the coronaviral and arteriviral mechanism, wherein RNA structures are insufficient and an accompanying body TRS is required to stimulate template switching. We cannot unambiguously identify which nucleotides are templated before or after the event, as a GUUU sequence maps to genomic RNA on either side of the breakpoint.

The leader-TRS chimeric reads mapping to the N protein gene initially appear consistent with the coronaviral and arteriviral mechanism of TRS-driven discontinuous RNA synthesis. However, close inspection indicated that the homologous motif mediating copy-choice recombination-like translocation and repairing of RNA strands was actually a short AGAA sequence, not the true TRS (tetranucleotides underlined in Fig. 15A and G). This would result in the nascent anti-TRS mispairing with the leader TRS, where two nucleotides are skipped once reinitiation occurs. This may explain why the discontinuous mechanism is utilized so rarely for this mRNA.

This leads to the suggestion that homology between any two sites is sufficient to induce template switching, i.e., that provided adequate sequence homology exists, the nascent RNA strand repairs with upstream sites within the genomic RNA regardless of the presence of a predefined TRS. This is consistent with the 5′ UTR-ORF1b chimeric transcripts, which again revealed a particular sequence that could be templated from either region in this case AACCUUA rather than the TRS.

If TRS sequence specificity is not required to stimulate EToV discontinuous RNA synthesis, it is presumably constrained by alternative roles. The highly conserved nature of the canonical leader, M, HE, and N TRSs (CUUUAG[A/U]) across all torovirus genomes (Fig. 15) suggests it is not tolerant to mutations; however, this has not been formally confirmed. Lack of conservation of the EToV U1, ORF1a, and ORF1b TRS sequences is consistent with them not being functionally relevant. Our results indicate this essential nature is likely due to a role in termination of RNA synthesis, as we did not identify a significant role of this motif in the generation of chimeric transcripts. Conversely, the upstream region of the extended TRS (cACN_3–4_CUUUAGA) is tolerant to modifications; indeed, even when this spacer is extended to six nucleotides, transcripts are still detectable at 20% of wild-type levels (36). Again, this is consistent with a role in termination rather than a requirement for repairing with upstream sequences. The canonical TRS sequences also presumably contribute to subgenomic promoter recognition, as the initial CAC is essential, although the adenylate is the first nucleotide on all positive-strand subgenomic transcripts (36). Initiation of sgRNA transcription at AC dinucleotides is also found in the roniviruses (12). It may be that in the Torovirinae and the Roniviridae, the conserved TRS is utilized primarily for signaling termination of RNA synthesis followed by promoter recognition, and any involvement in template switching is merely a by-product of RdRp promiscuity that has been coopted for gene expression in other nidovirids.

The unique combination of discontinuous and nondiscontinuous mechanisms within the one virus so far appears unique to the mammalian toroviruses. The one bafinivirus isolated to date (white bream virus, family Coronaviridae, subfamily Torovirinae, genus Bafinivirus) has an extended TRS sequence (CA[G/A]CACUAC) which is not conserved with the mammalian toroviruses analyzed in this study. Bafinivirus replication produces three sgRNAs which share an identical 42-nt leader also found at the far 5′ terminus of the genome, indicating this species utilizes discontinuous RNA synthesis in a manner similar to that of the corona- and arteriviruses (37). However, there was preliminary evidence that two of the three sgRNAs exhibit diversity in their junction sites, suggesting the anti-TRS binds to alternative sites within the 5′ leader during strand transfer, consistent with suggestions that, while a threshold level of homology is required, this is not limited to particular primary sequences (38, 39). This is reflected in the fact that the bafinivirus leader TRS is not fully identical to the body TRSs.

It is not known which mechanism was utilized by the last common ancestor of nidovirids and, thus, which represents divergence from the original model. It has been suggested that convergent evolution has resulted in the mechanism for discontinuous negative-strand synthesis arising multiple times within the Nidovirales (40). Similarly, whether the initial role of the TRS motif was to merely stimulate the attenuation of RNA synthesis or to direct template switching is not known. Our data suggest that transcription mechanisms in the Nidovirales fall into multiple categories, each requiring a distinct role of the TRS: (i) homology-driven reinitiation (canonical discontinuous RNA synthesis, as seen in coronaviruses and arteriviruses and, to a low extent, EToV N protein-coding mRNAs), (ii) structure-driven discontinuous transcription (EToV S gene), and (iii) transcription termination (EToV M, HE, and the majority of N protein-coding transcripts). These mechanisms require an RdRp which is prone to template switching when even relatively short homologous sequences are present, potentially leading to a large number of irrelevant transcripts being produced (as previously observed in an arterivirus [41, 42]) and also facilitating the production of defective interfering RNAs (36) and recombinant strains (7).

### Effects upon the host: transcriptional and translational differential expression.

The differential transcription analysis indicated that infection with EToV induces increased transcription of multiple genes, the products of which are significantly more likely than random to be involved in (i) nucleosome function and DNA binding and (ii) immune responses to infection than genes which were not differentially transcribed. Some of the identified GO categories, including cytokine signaling, innate immune responses, and ribosome biogenesis, have been identified in previous RNA-seq analyses of various coronaviruses (43, 44). Similarly, although differential translational analyses or proteomic studies have not been conducted on toroviruses, some of the identified proteins have been recognized as being incorporated into nidovirid virions (for example, TCP-1 and multiple heat shock proteins within arterivirus particles) (45). Others have been identified as being upregulated upon infection with coronaviruses, such as the solute carrier family 25 members (46). Notably, both poly(C) and poly(A) binding proteins were preferentially translated in infected cells; these have been previously identified as interaction partners of arteriviral nonstructural protein 1β and contribute to viral RNA replication (47). It therefore appears that torovirus infection induces a host response similar to that of many nidovirids.

To the best of our knowledge, this is the first analysis of differential gene expression following infection with a torovirus. It would be of interest to repeat this analysis at later time points, as a previous study found that EToV-mediated global inhibition of host protein synthesis was only detectable at 16 h postinfection (h.p.i.). The same study found induction of both the intrinsic and extrinsic apoptotic pathways was evident only by 24 h.p.i. (48). It is clear that the transcriptional and translational profile of the host cell may differ significantly throughout the course of infection. Additionally, it must be noted that the horse (Equus caballus) genome is not highly annotated, and thus many Ensembl gene identifications do not possess an annotated orthologue, a limiting factor in our analysis.

### What is the function of U1 and U2?

The current lack of a published reverse genetics system to study torovirus replication means we are unable to perform targeted mutagenesis. This would enable definitive experimental confirmation that U1 and U2 are translated from their respective CUG codons, followed by phenotypic analysis of knockout mutants. However, the comparative genomic analysis together with the accumulation of ribosomes on both CUG codons is highly suggestive of this being the site of initiation; CUG has previously been reported as the most commonly utilized non-AUG initiation codon in mammalian systems (49). In the case of U1, the coding sequence contains no AUG codons (in any frame), a situation that would facilitate preinitiation ribosomes to continue scanning to the U2 CUG and the ORF1a AUG initiation sites (50). It remains a possibility that U2 translation initiates at a downstream AUG; however, the only in-frame AUG is located 336 nt downstream of our presumed start site and is in a poor initiation context (C at −3) and 3′ of the ORF1a AUG. We are therefore confident that the CUG codons that were identified in the ribosome profiling data represent the genuine translational start sites.

The ORFs of both U1 and U2 are intact in all torovirus genomic sequences that we have analyzed to date, including bovine (51, 52), caprine, and porcine isolates (53). Most of the U2 ORF is constrained by the fact that the sequence must also retain ORF1a coding capacity in another frame. U1 is not under such limitations, although it is likely that the viral genome must maintain specific 5′ UTR structures to facilitate viral replication. Previous investigations utilizing defective interfering RNAs have confirmed that no more than the first 604 nt of the 5′ UTR and the entirety of the 3′ UTR are sufficient to allow both positive- and negative-strand RNA synthesis (36); it is notable that this region only includes one-third of the U1 ORF (which starts at nucleotide 524), hence only this subdomain would be constrained by maintaining two distinct functional roles. We suggest that the so-called 5′ UTR is actually limited to 523 nt preceding the CUG of U1, and the remainder of U1 and U2 is not under pressure to maintain *cis*-replication elements.

Neither ORF could be identified within the white bream virus genome, a bafinivirus that constitutes another genus within the subfamily Torovirinae (37), although the lack of multiple bafinivirus sequences makes comparative genomic analysis impossible.

The function(s) of the proteins encoded by both U1 and U2 remain to be elucidated. Despite the relatively large size of the U2 protein (∼30 kDa), after extensive database searches no structural homologs were identified. By comparison, the U1 protein is small (∼10 kDa) and highly basic (pI of 10.4), and it possesses many of the predicted features of a double-spanning transmembrane protein, including two hydrophobic stretches separated by a hinge and a predicted coiled-coil tertiary topology. Based on structural similarity to known proteins, one potential function is a virally encoded ion channel (viroporin) embedded in either intracellular or plasma membranes. It is possible that U1 plays a role in toroviruses similar to that of the coronaviral and arteriviral E proteins, which have no known toroviral homologue. The coronavirus E protein is a small transmembrane protein (∼10 kDa) which possesses ion channel activity and is required for virion assembly, forming a pentamer that traverses the viral envelope (54, 55). E proteins also possess a membrane-proximal palmitoylated cysteine residue, which is a predicted (and conserved) posttranslational modification for U1 (34).

Alternatively, viroporin activity may be mediated by a small, basic double-transmembrane protein, the ORF of which is embedded within the EToV N gene in the +1 frame (with respect to N). An analogous “N + 1” protein has been identified in some group II coronaviruses and is postulated to play a structural role; however, it is not essential for replication (56, 57). Neither our ribosome profiling nor comparative genomic analysis provides evidence that this ORF is utilized in toroviruses. We did not observe ribosomes translating in this frame in either the initial data set or the drug-treated samples (although Ribo-seq may not always detect poorly translated overlapping genes); further, the ORF is not preserved in all torovirus genomes.

Our data have revealed that the transcriptional landscape of a prototypic torovirus is complex and driven by many factors beyond the canonical multilocus TRS model of coronaviruses. The development of a torovirus reverse genetics system would allow manipulation of potential template switch-inducing sequences and allow us to elucidate which features of the toroviral TRS cause them to act as terminators of RNA synthesis rather than consistently inducing homology-assisted recombination. Our accompanying translational analysis has revealed two conserved novel ORFs and suggests the EToV 5′ UTR is only 523 nt. Together these data provide insight into the molecular biology of the replication cycle of this neglected pathogen and highlight the disparities between the families of the Nidovirales.

## MATERIALS AND METHODS

### Virus isolates.

A plaque-purified isolate of equine torovirus, Berne strain (isolate P138/72) (EToV), was kindly provided by Raoul de Groot (Utrecht University) and cultured in equine dermis (ED) cells. This virus was initially isolated from a symptomatic horse in 1972 (15). ED cells were maintained in Dulbecco's modified Eagle's medium (Invitrogen) supplemented with 10% fetal calf serum, 100 IU/ml penicillin, 100 μg/ml streptomycin, 1 mM nonessential amino acids, 25 mM HEPES, and 1% l-glutamine in a humidified incubator at 37°C with 5% CO_2_.

### RNA sequencing and ribosome profiling.

ED cells were infected with EToV for 1 h in serum-free medium (MOI of 0.1) and flash-frozen in liquid nitrogen at 8 h.p.i. prior to either RNA isolation or ribosome purification for profiling. Cells were either not pretreated or, where stated, were treated with a final concentration of 100 μg/ml cycloheximide (CHX) for 2 min (Sigma-Aldrich) or 2 μg/ml of harringtonine for 3 min (LKT Laboratories), followed by CHX for 2 min before flash-freezing. RNA and ribosomes were harvested according to previously published protocols (17, 58), with minor modifications. Following either RPF or RNA isolation, duplex-specific nuclease was not utilized but instead rRNA was depleted with the RiboZero [human/mouse/rat] kit (Illumina). Libraries were prepared and sequenced using the NextSeq500 platform (Illumina).

### Bioinformatic analysis of Ribo-seq and RNA-seq data.

Both Ribo-seq and RNA-seq reads were demultiplexed and adaptor sequences trimmed using the FASTX-Toolkit (http://hannonlab.cshl.edu/fastx_toolkit/). Reads shorter than 25 nt after trimming were discarded. Bowtie (version 1.2.1.1) databases were generated as follows. Horse rRNA sequences were downloaded from the National Center for Biotechnology Information (NCBI) Entrez Nucleotide database (accession numbers EU081775.1, NR_046271.1, NR_046309.2, EU554425.1, XM_014728542.1 and FN402126.1) (59). As the full-length virus RNA (vRNA) reference genome was not available for EToV, a reference was constructed from the following overlapping segments available from the Entrez Nucleotide database: DQ310701.1 (positions 1 to 14531), X52374.1 (13475 to 21394), X52506.1 (21250 to 26086), X52505.1 (26054 to 26850), X52375.1 (26784 to 27316), and D00563.1 (27264 to 279923). Horse mRNA sequences from EquCab2.0 (GCF_000002305.2) were downloaded from NCBI RefSeq (60). Horse ncRNA sequences were obtained from Ensembl release 89 (61) and combined with horse tRNA sequences from GtRNADB (62). Horse gDNA was obtained from Ensembl release 89. All horse sequences were from the EquCab2.0 genome build. Trimmed reads were then mapped sequentially to the rRNA, vRNA, mRNA, and ncRNA databases using bowtie, version 1.2.1.1 (63), with parameters –v 2 –best (i.e., maximum 2 mismatches, report best match), with only unmapped reads passed to each following stage. Reads that did not align to any of the aforementioned databases were then mapped to the host gDNA using STAR, version 2.5.4a (64), again allowing a maximum of 2 mismatches per alignment. Remaining reads were classified as unmapped.

Ribo-seq density and RNA-seq density were calculated for each gene in the EToV genome (Fig. 3 and 12). To normalize for different library sizes, reads per million mapped reads (RPM) values were calculated using the sum of positive-sense virus RNA reads and host RefSeq mRNA reads as the denominator. In order to standardize the regions used to calculate RNA-seq and Ribo-seq density, the following regions were selected: ORF1a, start codon (position 882) to 5′ end of frameshift site (position 14518); ORF1b, 3′ end of frameshift site (position 14525) to 5′ end of the S gene hairpin (position 21118); all other ORFs, initiation codon to termination codon. For U2, a region overlapping ORF1a was used because only 46 bases are unique to U2. For Fig. 12, the ORF1a coordinates were updated to exclude the region which overlaps U2, giving a range from 1552 to 21394. In addition, for all ORFs, only Ribo-seq reads mapping to the predominant phase (i.e., reads mapping to the first positions of codons) were used, as this should greatly diminish misassignment of ORF1a-translating ribosomes to U2 or *vice versa*. Reads mapping to the first five codons at the 5′ end of each region or the last six codons at the 3′ end of each region were excluded. For sgRNAs, RNA-seq density was calculated for the same regions as those described for Ribo-seq. For the genomic RNA the regions for ORF1a and ORF1b were combined into the interval from the start codon of ORF1a (position 882) to the 5′ end of the S gene hairpin (position 21118). Ribo-seq and RNA-seq densities were calculated as the RPM values for which the 5′ end maps to each region, divided by the length of the region in nucleotides and then multiplied by 1,000 (i.e., RPKM). For RNA-seq, a decumulation strategy was used to subtract the estimated RNA-seq density for longer overlapping genomic and subgenomic transcripts that would contribute to the RNA-seq density measured for each of the 3′ ORFs: the genomic RNA-seq density was subtracted from all subgenomic densities, and then the RNA-seq densities of overlapping upstream subgenomic transcripts were iteratively subtracted from downstream regions (e.g., RNA-seq density in the unique region of M was subtracted from HE, and this was subtracted from N). Translation efficiency for each gene was calculated as Ribo-seq density/decumulated RNA-seq density. Translational efficiencies for HE could not be accurately estimated, as the low expression of the HE transcript made the decumulation procedure for HE susceptible to noise.

Read length distributions were calculated for Ribo-seq and RNA-seq reads mapping to positive-sense host mRNA annotated coding DNA sequences (CDSs) or to the positive-sense coding sequence of the EToV genome (Fig. 4). Histograms of host mRNA Ribo-seq and RNA-seq 5′-end positions relative to initiation and termination codons (Fig. 5 and 6) were derived from reads mapping to mRNAs with annotated CDSs of ≥450 nt in length and annotated 5′ and 3′ UTRs of ≥60 nt in length. Host mRNA Ribo-seq and RNA-seq phasing distribution (Fig. 7) calculations took into account interior regions of annotated coding ORFs only (specifically, reads for which the 5′ end mapped between the first nucleotide of the initiation codon and 30 nt 5′ of the termination codon) in order to exclude reads on or near initiation or termination codons. For viral genome coverage plots, but not for meta-analyses of host RefSeq mRNA coverage, mapping positions of RPF 5′ ends were offset +12 nt to approximate the location of the ribosomal P-site (17).

### Analysis of viral transcripts.

The EToV (Berne isolate) genome sequence was confirmed by *de novo* assembly of unmapped and vRNA reads from the infected RNA-seq samples. Assembly was performed using Trinity (65) with the default settings for stranded single-ended (–SS_lib type “F”) data. Viral contigs were identified using BLASTN (29) against a database of EToV reference sequences based on the NCBI records listed above. The viral contig was aligned to the reference using the MAFFT L-INS-i method (66).

Chimeric reads were classified as reads for which the entire read mapped uniquely to the viral genome, with no mismatches, after adding a single breakpoint, with a minimum of 12 nt mapping on either side of the breakpoint, at least 5 nt apart. To identify such reads, all unmapped reads were split into two subreads at every possible position ≥12 nt from either end, and these subreads were mapped to the viral genome using bowtie with no mismatches and no multimapping permitted. Transcription junctions were defined as donor/acceptor pairs that were either supported by at least 10 chimeric reads or contained the entire 5′ leader and TRS sequence in the 5′ segment of the read. At some positions single-nucleotide resolution for the chimeric breakpoint could not be established. Where reads were found to break at adjacent possible positions, these positions were merged to give a short region containing the breakpoint. The number of nonchimeric reads spanning each donor and acceptor site was calculated as the number of reads which overlapped the site by at least 12 nt in either direction (as chimeric reads overlapping the site by <12 nt are not detectable). The proportion of chimeric reads at each donor or acceptor site is therefore the number of chimeric reads with a breakpoint at the site divided by this number plus the number of nonchimeric reads spanning the site (Fig. 8B).

To visualize TRS conservation, multiple-sequence alignments were generated using Clustal Omega with default parameters (67). RNA structure was predicted using RNA-Alifold (68) and visualized using VARNA (69).

### Differential gene expression analysis.

For analysis of host differential expression between non-drug-treated infected and mock-infected cells, all reads which did not map to rRNA or vRNA were mapped to the EquCab2.0 reference genome and annotations (Ensembl release 89) using STAR (64), with a maximum of two mismatches and removal of noncanonical, nonannotated splice junctions. Read counts were generated using HTSeq 0.8.0 (70). For differential transcription analysis, gene level counts were generated across the Ensembl release 89 EquCab2.0 gtf file, filtered to include only protein-coding genes. For differential translation efficiency analysis, only coding regions (CDS) were considered: both RNA-seq and Ribo-seq counts were generated at the CDS level using intersection-strict mode, based on the same annotation set. Multimapping reads were excluded from both analyses. Differential transcript abundance analysis was performed using the standard DESeq2 (71) pipeline described in the vignette. Genes for which <10 reads were mapped were discarded. All recommended quality control plots were inspected, and no major biases were identified in the data. False discovery rate (FDR) values were calculated using the R fdrtool package (72). Genes with a log_2_ fold change of >1 and an FDR of less than 0.1 were considered to be differentially expressed. Gene ontology (GO) term enrichment analysis (73) was performed against a background of all horse protein-coding genes in the Ensembl gtf using a Fisher exact test and corrected for multiple testing with a Bonferroni correction. GO annotations for horse genes were downloaded from BiomaRt (Ensembl release 90) (74). Differential translational efficiency analysis was carried out using the CDS counts table, normalized using the DESeq2 “sizeFactors” technique. Similar to the differential transcription analysis, genes to which <10 reads mapped were discarded. All recommended quality control plots for DESeq2 were inspected again, and no major biases were identified in the data. Differential translation efficiency analysis was performed using Xtail (75) by following the standard pipeline described in the vignette. *P* values were adjusted automatically within Xtail using the Benjamini–Hochberg method. Genes with a log_2_ fold change of >1 and an adjusted *P* value of less than 0.1 were considered to be differentially translated. GO enrichment analysis was performed as described for the differential transcript abundance analysis.

### Comparative genomics.

The GenBank accession numbers utilized for comparative genomic analysis were DQ310701.1 (Berne virus), AY427798.1 (Breda virus) (51), KR527150.1 (goat torovirus), JQ860350.1 (porcine torovirus) (53), KM403390.1 (porcine torovirus) (76), LT900503.1 (porcine torovirus), LC088094.1 (bovine torovirus), and LC088095.1 (bovine torovirus) (52). *dN*/*dS* was estimated using the codeml program in the PAML package (24). The eight torovirus U1 nucleotide sequences were translated and aligned as amino acids with MUSCLE (77), and the amino acid alignment was used to guide a codon-based nucleotide alignment (EMBOSS tranalign) (78). Alignment columns with gap characters in any sequence were removed, resulting in a reduction from 81 to 79 codon positions. PhyML (79) was used to produce a nucleotide phylogenetic tree for the U1 alignment, and using this tree topology, *dN*/*dS* was calculated with codeml. The standard deviations for the codeml *dN*/*dS* value was estimated via a bootstrapping procedure in which codon columns of the alignment were randomly resampled (with replacement); 100 randomized alignments were generated, and their *dN*/*dS* values were calculated with codeml.

Coding potential within each reading frame was analyzed using MLOGD (25), and synonymous site conservation was analyzed with synplot2 (31). For these analyses we generated a codon-respecting alignment of the eight torovirus full-genome sequences using a procedure described previously (31). In brief, each individual genome sequence was aligned to a reference sequence using code2aln version 1.2 (80). Breda virus (GenBank accession number AY427798) was used as a reference, since unlike Berne virus it contains an intact HE gene. Genomes were then mapped to reference sequence coordinates by removing alignment positions that contained a gap character in the reference sequence, and these pairwise alignments were combined to give the multiple-sequence alignment. This was analyzed with MLOGD using a 40-codon sliding window and a 5-codon step size. For each of the three reading frames, within each window the null model is that the sequence is noncoding, whereas the alternative model is that the sequence is coding in the given reading frame. Positive/negative values indicate that the sequences in the alignment are likely/unlikely to be coding in the given reading frame. To assess conservation at synonymous sites, the concatenated coding regions were extracted from the alignment and analyzed with synplot2.

### Data availability.

The sequencing data reported in this paper have been deposited in ArrayExpress (http://www.ebi.ac.uk/arrayexpress) under the accession number E-MTAB-6656. The full-length virus sequence has been deposited in GenBank (accession no. MG996765).

## Supplementary Material

Supplemental file 1

Supplemental file 2

## References

[1] Lauber C, Ziebuhr J, Junglen S, Drosten C, Zirkel F, Nga PT, Morita K, Snijder EJ, Gorbalenya AE 2012 Mesoniviridae: a proposed new family in the order Nidovirales formed by a single species of mosquito-borne viruses. Arch Virol 157:1623–1628. doi:10.1007/s00705-012-1295-x. 22527862 PMC3407358

[2] Hoet AE, Saif LJ 2004 Bovine torovirus (Breda virus) revisited. Anim Health Res Rev 5:157–171. doi:10.1079/AHR200498. 15984322

[3] Brown DW, Beards GM, Flewett TH 1987 Detection of Breda virus antigen and antibody in humans and animals by enzyme immunoassay. J Clin Microbiol 25:637–640. 3571473 10.1128/jcm.25.4.637-640.1987 PMC266050

[4] Alonso-Padilla J, Pignatelli J, Simon-Grife M, Plazuelo S, Casal J, Rodriguez D 2012 Seroprevalence of porcine torovirus (PToV) in Spanish farms. BMC Res Notes 5:675. doi:10.1186/1756-0500-5-675. 23217216 PMC3556161

[5] Hanke D, Pohlmann A, Sauter-Louis C, Hoper D, Stadler J, Ritzmann M, Steinrigl A, Schwarz BA, Akimkin V, Fux R, Blome S, Beer M 2017 Porcine epidemic diarrhea in Europe: in-detail analyses of disease dynamics and molecular epidemiology. Viruses 9:E177. doi:10.3390/v9070177. 28684708 PMC5537669

[6] Pignatelli J, Grau-Roma L, Jimenez M, Segales J, Rodriguez D 2010 Longitudinal serological and virological study on porcine torovirus (PToV) in piglets from Spanish farms. Vet Microbiol 146:260–268. doi:10.1016/j.vetmic.2010.05.023. 20542392 PMC7127003

[7] Smits SL, Lavazza A, Matiz K, Horzinek MC, Koopmans MP, de Groot RJ 2003 Phylogenetic and evolutionary relationships among torovirus field variants: evidence for multiple intertypic recombination events. J Virol 77:9567–9577. doi:10.1128/JVI.77.17.9567-9577.2003. 12915570 PMC187415

[8] Sawicki SG, Sawicki DL, Siddell SG 2007 A contemporary view of coronavirus transcription. J Virol 81:20–29. doi:10.1128/JVI.01358-06. 16928755 PMC1797243

[9] Sola I, Almazan F, Zuniga S, Enjuanes L 2015 Continuous and discontinuous RNA synthesis in coronaviruses. Annu Rev Virol 2:265–288. doi:10.1146/annurev-virology-100114-055218. 26958916 PMC6025776

[10] van Marle G, Dobbe JC, Gultyaev AP, Luytjes W, Spaan WJ, Snijder EJ 1999 Arterivirus discontinuous mRNA transcription is guided by base pairing between sense and antisense transcription-regulating sequences. Proc Natl Acad Sci U S A 96:12056–12061. doi:10.1073/pnas.96.21.12056. 10518575 PMC18411

[11] Pasternak AO, Spaan WJ, Snijder EJ 2006 Nidovirus transcription: how to make sense? J Gen Virol 87:1403–1421. doi:10.1099/vir.0.81611-0. 16690906

[12] Cowley JA, Dimmock CM, Walker PJ 2002 Gill-associated nidovirus of Penaeus monodon prawns transcribes 3′-coterminal subgenomic mRNAs that do not possess 5′-leader sequences. J Gen Virol 83:927–935. doi:10.1099/0022-1317-83-4-927. 11907343

[13] Zirkel F, Roth H, Kurth A, Drosten C, Ziebuhr J, Junglen S 2013 Identification and characterization of genetically divergent members of the newly established family Mesoniviridae. J Virol 87:6346–6358. doi:10.1128/JVI.00416-13. 23536661 PMC3648093

[14] van Vliet AL, Smits SL, Rottier PJ, de Groot RJ 2002 Discontinuous and non-discontinuous subgenomic RNA transcription in a nidovirus. EMBO J 21:6571–6580. doi:10.1093/emboj/cdf635. 12456663 PMC136939

[15] Weiss M, Steck F, Horzinek MC 1983 Purification and partial characterization of a new enveloped RNA virus (Berne virus). J Gen Virol 64(Part 9):1849–1858. doi:10.1099/0022-1317-64-9-1849. 6886677

[16] Kuwabara M, Wada K, Maeda Y, Miyazaki A, Tsunemitsu H 2007 First isolation of cytopathogenic bovine torovirus in cell culture from a calf with diarrhea. Clin Vaccine Immunol 14:998–1004. doi:10.1128/CVI.00475-06. 17567770 PMC2044491

[17] Irigoyen N, Firth AE, Jones JD, Chung BY, Siddell SG, Brierley I 2016 High-resolution analysis of coronavirus gene expression by RNA sequencing and ribosome profiling. PLoS Pathog 12:e1005473. doi:10.1371/journal.ppat.1005473. 26919232 PMC4769073

[18] Snijder EJ, den Boon JA, Horzinek MC, Spaan WJ 1991 Comparison of the genome organization of toro- and coronaviruses: evidence for two nonhomologous RNA recombination events during Berne virus evolution. Virology 180:448–452. doi:10.1016/0042-6822(91)90056-H. 1984666 PMC7126633

[19] Snijder EJ, den Boon JA, Bredenbeek PJ, Horzinek MC, Rijnbrand R, Spaan WJ 1990 The carboxyl-terminal part of the putative Berne virus polymerase is expressed by ribosomal frameshifting and contains sequence motifs which indicate that toro- and coronaviruses are evolutionarily related. Nucleic Acids Res 18:4535–4542. doi:10.1093/nar/18.15.4535. 2388833 PMC331274

[20] Yang D, Leibowitz JL 2015 The structure and functions of coronavirus genomic 3′ and 5′ ends. Virus Res 206:120–133. doi:10.1016/j.virusres.2015.02.025. 25736566 PMC4476908

[21] Snijder EJ, den Boon JA, Horzinek MC, Spaan WJ 1991 Characterization of defective interfering RNAs of Berne virus. J Gen Virol 72(Part 7):1635–1643. doi:10.1099/0022-1317-72-7-1635. 1856694

[22] Gerashchenko MV, Gladyshev VN 2014 Translation inhibitors cause abnormalities in ribosome profiling experiments. Nucleic Acids Res 42:e134. doi:10.1093/nar/gku671. 25056308 PMC4176156

[23] Andreev DE, O'Connor PB, Loughran G, Dmitriev SE, Baranov PV, Shatsky IN 2017 Insights into the mechanisms of eukaryotic translation gained with ribosome profiling. Nucleic Acids Res 45:513–526. doi:10.1093/nar/gkw1190. 27923997 PMC5314775

[24] Yang Z 2007 PAML 4: phylogenetic analysis by maximum likelihood. Mol Biol Evol 24:1586–1591. doi:10.1093/molbev/msm088. 17483113

[25] Firth AE, Brown CM 2006 Detecting overlapping coding sequences in virus genomes. BMC Bioinformatics 7:75. doi:10.1186/1471-2105-7-75. 16483358 PMC1395342

[26] Firth AE, Atkins JF 2009 A case for a CUG-initiated coding sequence overlapping torovirus ORF1a and encoding a novel 30 kDa product. Virol J 6:136. doi:10.1186/1743-422X-6-136. 19737402 PMC2749830

[27] Keese PK, Gibbs A 1992 Origins of genes: “big bang” or continuous creation? Proc Natl Acad Sci U S A 89:9489–9493. doi:10.1073/pnas.89.20.9489. 1329098 PMC50157

[28] Rancurel C, Khosravi M, Dunker AK, Romero PR, Karlin D 2009 Overlapping genes produce proteins with unusual sequence properties and offer insight into de novo protein creation. J Virol 83:10719–10736. doi:10.1128/JVI.00595-09. 19640978 PMC2753099

[29] Altschul SF, Gish W, Miller W, Myers EW, Lipman DJ 1990 Basic local alignment search tool. J Mol Biol 215:403–410. doi:10.1016/S0022-2836(05)80360-2. 2231712

[30] Soding J, Biegert A, Lupas AN 2005 The HHpred interactive server for protein homology detection and structure prediction. Nucleic Acids Res 33:W244–W248. doi:10.1093/nar/gki408. 15980461 PMC1160169

[31] Firth AE 2014 Mapping overlapping functional elements embedded within the protein-coding regions of RNA viruses. Nucleic Acids Res 42:12425–12439. doi:10.1093/nar/gku981. 25326325 PMC4227794

[32] Kozak M 1986 Point mutations define a sequence flanking the AUG initiator codon that modulates translation by eukaryotic ribosomes. Cell 44:283–292. doi:10.1016/0092-8674(86)90762-2. 3943125

[33] Haas J, Roth S, Arnold K, Kiefer F, Schmidt T, Bordoli L, Schwede T 2013 The Protein Model Portal–a comprehensive resource for protein structure and model information. Database (Oxford) 2013:bat031. doi:10.1093/database/bat031. 23624946 PMC3889916

[34] Kelley LA, Mezulis S, Yates CM, Wass MN, Sternberg MJ 2015 The Phyre2 web portal for protein modeling, prediction and analysis. Nat Protoc 10:845–858. doi:10.1038/nprot.2015.053. 25950237 PMC5298202

[35] McGuffin LJ, Bryson K, Jones DT 2000 The PSIPRED protein structure prediction server. Bioinformatics 16:404–405. doi:10.1093/bioinformatics/16.4.404. 10869041

[36] Smits SL, van Vliet AL, Segeren K, el Azzouzi H, van Essen M, de Groot RJ 2005 Torovirus non-discontinuous transcription: mutational analysis of a subgenomic mRNA promoter. J Virol 79:8275–8281. doi:10.1128/JVI.79.13.8275-8281.2005. 15956573 PMC1143767

[37] Schutze H, Ulferts R, Schelle B, Bayer S, Granzow H, Hoffmann B, Mettenleiter TC, Ziebuhr J 2006 Characterization of White bream virus reveals a novel genetic cluster of nidoviruses. J Virol 80:11598–11609. doi:10.1128/JVI.01758-06. 16987966 PMC1642614

[38] Sola I, Alonso S, Zuniga S, Balasch M, Plana-Duran J, Enjuanes L 2003 Engineering the transmissible gastroenteritis virus genome as an expression vector inducing lactogenic immunity. J Virol 77:4357–4369. doi:10.1128/JVI.77.7.4357-4369.2003. 12634392 PMC150661

[39] Zuniga S, Sola I, Alonso S, Enjuanes L 2004 Sequence motifs involved in the regulation of discontinuous coronavirus subgenomic RNA synthesis. J Virol 78:980–994. doi:10.1128/JVI.78.2.980-994.2004. 14694129 PMC368802

[40] de Vries AAF, Horzinek MC, Rottier PJM, de Groot RJ 1997 The genome organization of the Nidovirales: similarities and differences between arteri-, toro-, and coronaviruses. Semin Virol 8:33–47. doi:10.1006/smvy.1997.0104. PMC7128191 32288441

[41] Di H, McIntyre AA, Brinton MA 2018 New insights about the regulation of Nidovirus subgenomic mRNA synthesis. Virology 517:38–43. doi:10.1016/j.virol.2018.01.026. 29475599 PMC5987246

[42] Di H, Madden JC Jr, Morantz EK, Tang HY, Graham RL, Baric RS, Brinton MA 2017 Expanded subgenomic mRNA transcriptome and coding capacity of a nidovirus. Proc Natl Acad Sci U S A 114:E8895–E8904. doi:10.1073/pnas.1706696114. 29073030 PMC5651751

[43] Cong F, Liu X, Han Z, Shao Y, Kong X, Liu S 2013 Transcriptome analysis of chicken kidney tissues following coronavirus avian infectious bronchitis virus infection. BMC Genomics 14:743. doi:10.1186/1471-2164-14-743. 24168272 PMC3870970

[44] Raaben M, Groot Koerkamp MJ, Rottier PJ, de Haan CA 2007 Mouse hepatitis coronavirus replication induces host translational shutoff and mRNA decay, with concomitant formation of stress granules and processing bodies. Cell Microbiol 9:2218–2229. doi:10.1111/j.1462-5822.2007.00951.x. 17490409 PMC7162177

[45] Zhang C, Xue C, Li Y, Kong Q, Ren X, Li X, Shu D, Bi Y, Cao Y 2010 Profiling of cellular proteins in porcine reproductive and respiratory syndrome virus virions by proteomics analysis. Virol J 7:242. doi:10.1186/1743-422X-7-242. 20849641 PMC2949843

[46] VanLeuven JT, Ridenhour BJ, Gonzalez AJ, Miller CR, Miura TA 2017 Lung epithelial cells have virus-specific and shared gene expression responses to infection by diverse respiratory viruses. PLoS One 12:e0178408. doi:10.1371/journal.pone.0178408. 28575086 PMC5456070

[47] Beura LK, Dinh PX, Osorio FA, Pattnaik AK 2011 Cellular poly(c) binding proteins 1 and 2 interact with porcine reproductive and respiratory syndrome virus nonstructural protein 1beta and support viral replication. J Virol 85:12939–12949. doi:10.1128/JVI.05177-11. 21976648 PMC3233143

[48] Maestre AM, Garzon A, Rodriguez D 2011 Equine torovirus (BEV) induces caspase-mediated apoptosis in infected cells. PLoS One 6:e20972. doi:10.1371/journal.pone.0020972. 21698249 PMC3115971

[49] Touriol C, Bornes S, Bonnal S, Audigier S, Prats H, Prats AC, Vagner S 2003 Generation of protein isoform diversity by alternative initiation of translation at non-AUG codons. Biol Cell 95:169–178. doi:10.1016/S0248-4900(03)00033-9. 12867081

[50] Firth AE, Brierley I 2012 Non-canonical translation in RNA viruses. J Gen Virol 93:1385–1409. doi:10.1099/vir.0.042499-0. 22535777 PMC3542737

[51] Draker R, Roper RL, Petric M, Tellier R 2006 The complete sequence of the bovine torovirus genome. Virus Res 115:56–68. doi:10.1016/j.virusres.2005.07.005. 16137782 PMC7114287

[52] Ito M, Tsuchiaka S, Naoi Y, Otomaru K, Sato M, Masuda T, Haga K, Oka T, Yamasato H, Omatsu T, Sugimura S, Aoki H, Furuya T, Katayama Y, Oba M, Shirai J, Katayama K, Mizutani T, Nagai M 2016 Whole genome analysis of Japanese bovine toroviruses reveals natural recombination between porcine and bovine toroviruses. Infect Genet Evol 38:90–95. doi:10.1016/j.meegid.2015.12.013. 26708248 PMC7185535

[53] Sun H, Lan D, Lu L, Chen M, Wang C, Hua X 2014 Molecular characterization and phylogenetic analysis of the genome of porcine torovirus. Arch Virol 159:773–778. doi:10.1007/s00705-013-1861-x. 24122107 PMC7086580

[54] Nieto-Torres JL, DeDiego ML, Verdia-Baguena C, Jimenez-Guardeno JM, Regla-Nava JA, Fernandez-Delgado R, Castano-Rodriguez C, Alcaraz A, Torres J, Aguilella VM, Enjuanes L 2014 Severe acute respiratory syndrome coronavirus envelope protein ion channel activity promotes virus fitness and pathogenesis. PLoS Pathog 10:e1004077. doi:10.1371/journal.ppat.1004077. 24788150 PMC4006877

[55] Ruch TR, Machamer CE 2012 The coronavirus E protein: assembly and beyond. Viruses 4:363–382. doi:10.3390/v4030363. 22590676 PMC3347032

[56] Fischer F, Peng D, Hingley ST, Weiss SR, Masters PS 1997 The internal open reading frame within the nucleocapsid gene of mouse hepatitis virus encodes a structural protein that is not essential for viral replication. J Virol 71:996–1003. 8995618 10.1128/jvi.71.2.996-1003.1997 PMC191149

[57] Senanayake SD, Hofmann MA, Maki JL, Brian DA 1992 The nucleocapsid protein gene of bovine coronavirus is bicistronic. J Virol 66:5277–5283. 1501275 10.1128/jvi.66.9.5277-5283.1992 PMC289081

[58] Irigoyen N, Dinan AM, Brierley I, Firth AE 2018 Ribosome profiling of the retrovirus murine leukemia virus. Retrovirology 15:10. doi:10.1186/s12977-018-0394-5. 29357872 PMC5778647

[59] NCBI Resource Coordinators. 2016 Database resources of the National Center for Biotechnology Information. Nucleic Acids Res 44:D7–D19. doi:10.1093/nar/gkv1290. 26615191 PMC4702911

[60] Pruitt KD, Tatusova T, Brown GR, Maglott DR 2012 NCBI reference sequences (RefSeq): current status, new features and genome annotation policy. Nucleic Acids Res 40:D130–D135. doi:10.1093/nar/gkr1079. 22121212 PMC3245008

[61] Flicek P, Amode MR, Barrell D, Beal K, Brent S, Carvalho-Silva D, Clapham P, Coates G, Fairley S, Fitzgerald S, Gil L, Gordon L, Hendrix M, Hourlier T, Johnson N, Kahari AK, Keefe D, Keenan S, Kinsella R, Komorowska M, Koscielny G, Kulesha E, Larsson P, Longden I, McLaren W, Muffato M, Overduin B, Pignatelli M, Pritchard B, Riat HS, Ritchie GR, Ruffier M, Schuster M, Sobral D, Tang YA, Taylor K, Trevanion S, Vandrovcova J, White S, Wilson M, Wilder SP, Aken BL, Birney E, Cunningham F, Dunham I, Durbin R, Fernandez-Suarez XM, Harrow J, Herrero J, Hubbard TJ, Parker A, Proctor G, Spudich G, Vogel J, Yates A, Zadissa A, Searle SM 2012 Ensembl 2012. Nucleic Acids Res 40:D84–D90. doi:10.1093/nar/gkr991. 22086963 PMC3245178

[62] Chan PP, Lowe TM 2016 GtRNAdb 2.0: an expanded database of transfer RNA genes identified in complete and draft genomes. Nucleic Acids Res 44:D184–D189. doi:10.1093/nar/gkv1309. 26673694 PMC4702915

[63] Langmead B, Trapnell C, Pop M, Salzberg SL 2009 Ultrafast and memory-efficient alignment of short DNA sequences to the human genome. Genome Biol 10:R25. doi:10.1186/gb-2009-10-3-r25. 19261174 PMC2690996

[64] Dobin A, Davis CA, Schlesinger F, Drenkow J, Zaleski C, Jha S, Batut P, Chaisson M, Gingeras TR 2013 STAR: ultrafast universal RNA-seq aligner. Bioinformatics 29:15–21. doi:10.1093/bioinformatics/bts635. 23104886 PMC3530905

[65] Grabherr MG, Haas BJ, Yassour M, Levin JZ, Thompson DA, Amit I, Adiconis X, Fan L, Raychowdhury R, Zeng Q, Chen Z, Mauceli E, Hacohen N, Gnirke A, Rhind N, di Palma F, Birren BW, Nusbaum C, Lindblad-Toh K, Friedman N, Regev A 2011 Full-length transcriptome assembly from RNA-seq data without a reference genome. Nat Biotechnol 29:644–652. doi:10.1038/nbt.1883. 21572440 PMC3571712

[66] Katoh K, Standley DM 2013 MAFFT multiple sequence alignment software version 7: improvements in performance and usability. Mol Biol Evol 30:772–780. doi:10.1093/molbev/mst010. 23329690 PMC3603318

[67] Sievers F, Higgins DG 2014 Clustal omega. Curr Protoc Bioinformatics 48:3.13.1–3.13.16. 10.1002/0471250953.bi0313s48 25501942

[68] Bernhart SH, Hofacker IL, Will S, Gruber AR, Stadler PF 2008 RNAalifold: improved consensus structure prediction for RNA alignments. BMC Bioinformatics 9:474. doi:10.1186/1471-2105-9-474. 19014431 PMC2621365

[69] Darty K, Denise A, Ponty Y 2009 VARNA: Interactive drawing and editing of the RNA secondary structure. Bioinformatics 25:1974–1975. doi:10.1093/bioinformatics/btp250. 19398448 PMC2712331

[70] Anders S, Pyl PT, Huber W 2015 HTSeq–a Python framework to work with high-throughput sequencing data. Bioinformatics 31:166–169. doi:10.1093/bioinformatics/btu638. 25260700 PMC4287950

[71] Love MI, Huber W, Anders S 2014 Moderated estimation of fold change and dispersion for RNA-seq data with DESeq2. Genome Biol 15:550. doi:10.1186/s13059-014-0550-8. 25516281 PMC4302049

[72] Strimmer K 2008 fdrtool: a versatile R package for estimating local and tail area-based false discovery rates. Bioinformatics 24:1461–1462. doi:10.1093/bioinformatics/btn209. 18441000

[73] Ashburner M, Ball CA, Blake JA, Botstein D, Butler H, Cherry JM, Davis AP, Dolinski K, Dwight SS, Eppig JT, Harris MA, Hill DP, Issel-Tarver L, Kasarskis A, Lewis S, Matese JC, Richardson JE, Ringwald M, Rubin GM, Sherlock G 2000 Gene ontology: tool for the unification of biology. The Gene Ontology Consortium. Nat Genet 25:25–29. 10802651 10.1038/75556 PMC3037419

[74] Durinck S, Spellman PT, Birney E, Huber W 2009 Mapping identifiers for the integration of genomic datasets with the R/Bioconductor package biomaRt. Nat Protoc 4:1184–1191. doi:10.1038/nprot.2009.97. 19617889 PMC3159387

[75] Xiao Z, Zou Q, Liu Y, Yang X 2016 Genome-wide assessment of differential translations with ribosome profiling data. Nat Commun 7:11194. doi:10.1038/ncomms11194. 27041671 PMC4822032

[76] Anbalagan S, Peterson J, Wassman B, Elston J, Schwartz K 2014 Genome sequence of torovirus identified from a pig with porcine epidemic diarrhea virus from the United States. Genome Announc 2:e01291-14. doi:10.1128/genomeA.01291-14. 25523767 PMC4271157

[77] Edgar RC 2004 MUSCLE: multiple sequence alignment with high accuracy and high throughput. Nucleic Acids Res 32:1792–1797. doi:10.1093/nar/gkh340. 15034147 PMC390337

[78] Rice P, Longden I, Bleasby A 2000 EMBOSS: the European Molecular Biology Open Software Suite. Trends Genet 16:276–277. doi:10.1016/S0168-9525(00)02024-2. 10827456

[79] Guindon S, Gascuel O 2003 A simple, fast, and accurate algorithm to estimate large phylogenies by maximum likelihood. Syst Biol 52:696–704. doi:10.1080/10635150390235520. 14530136

[80] Stocsits RR, Hofacker IL, Fried C, Stadler PF 2005 Multiple sequence alignments of partially coding Nucleic acid sequences. BMC Bioinformatics 6:160. doi:10.1186/1471-2105-6-160. 15985156 PMC1182351

